# Pharmacological restoration of impaired autophagy in retinal ganglion cells prevents abnormal mitochondrial accumulation and glaucomatous neurodegeneration

**DOI:** 10.1186/s13024-026-00950-4

**Published:** 2026-05-16

**Authors:** Prabhavathi Maddineni, Balasankara Reddy Kaipa, Bindu Kodati, Karthikeyan Kesavan, Linya Li, J Cameron Millar, Sam Yacoub, Ramesh B. Kasetti, Abbot F. Clark, Gulab S. Zode

**Affiliations:** 1 Department of Ophthalmology, School of Medicine, University of Missouri, 1 Hospital Dr, Columbia, MO 65212 USA; 2 Gavin Herbert Eye Institute-Center for Translational Vision Research, Department of Ophthalmology, Department of Physiology and Biophysics, Irvine School of Medicine, University of California, 829 Health Sciences Rd, Irvine, CA 92697 USA; 3 Department of Pharmacology and Neuroscience and North Texas Eye Research Institute, University of North Texas Health Science Center at Fort Worth, Fort Worth, TX 76107 USA

**Keywords:** Glaucoma, Intraocular pressure, Optic neuropathy, Mitophagy, Mitochondrial dysfunction, Neurodegeneration, Oxidative DNA damage, Mouse models of glaucoma, Torin 2, Autophagy

## Abstract

**Background:**

Progressive loss of retinal ganglion cells (RGCs) and degeneration of optic nerve (ON) axons are the key pathological hallmarks of glaucoma, the leading cause of irreversible blindness. Elevated intraocular pressure (IOP), primarily due to dysfunction of the trabecular meshwork (TM), remains the most significant and only known modifiable risk factor. However, vision loss persists in some patients despite effective IOP control, highlighting the critical need to elucidate the mechanisms driving glaucomatous neurodegeneration. Emerging evidence links mitochondrial dysfunction to glaucomatous neurodegeneration, yet the precise mechanisms remain poorly defined. Here, we investigate whether defective autophagy/mitophagy, which removes damaged mitochondria, contributes to mitochondrial accumulation, oxidative stress, and neurodegeneration in glaucoma. We further explore the therapeutic potential of enhancing autophagy to improve mitochondrial turnover, mitigate RGC loss, and preserve visual function.

**Methods:**

Glucocorticoid (GC)-induced and myocilin (MYOC)-associated glaucoma mouse models were used to assess the expression of mitochondrial markers (TOM20/COX IV), oxidative DNA damage (8-OHdG), and mitophagy/autophagy-related proteins (p62, LC3, Phospho-ubiquitin (Ser65), and LAMP1) in retinal tissues. Transmission electron microscopy (TEM) was employed to analyze mitochondrial accumulation in glaucomatous ON. Mitophagy flux was assessed at early and late stages of neurodegeneration using mitophagy reporter Mt-Keima mice. The effect of RGC-specific autophagy deficiency on mitochondrial accumulation and neurodegeneration was further investigated using Atg5^flox/flox^ mice, in which Atg5 deletion was induced by AAV2-Cre delivery. Additionally, the therapeutic effect of enhancing autophagy with Torin 2 to restore mitochondrial turnover and prevent glaucomatous neurodegeneration was evaluated in both GC-induced and myocilin-associated glaucoma models, as well as in ex vivo human retinal explants.

**Results:**

Chronic IOP elevation led to increased mitochondrial accumulation, oxidative DNA damage, and impaired mitophagy/autophagy in glaucomatous retina. TEM analysis further confirmed the accumulation of structurally abnormal mitochondria in glaucomatous ON. In Mt-Keima mice, chronic IOP elevation significantly reduced mitophagy flux prior to RGC loss, indicating that mitophagy impairment precedes neurodegeneration. RGC-specific Atg5 deletion induced the accumulation of damaged mitochondria, leading to neurodegeneration in Atg5 ^flox/flox^ mice. Notably, pharmacological restoration of impaired autophagy with Torin 2 prevented mitochondrial accumulation and preserved the structural and functional integrity of RGCs and their axons in glaucoma mouse models and ex vivo human retinal explant cultures.

**Conclusion:**

Our study indicates impaired autophagy contributes to damaged mitochondrial accumulation and oxidative stress, leading to glaucomatous neurodegeneration. Enhancing autophagy in RGCs represents a promising therapeutic strategy to prevent glaucomatous neurodegeneration.

**Graphical Abstract:**

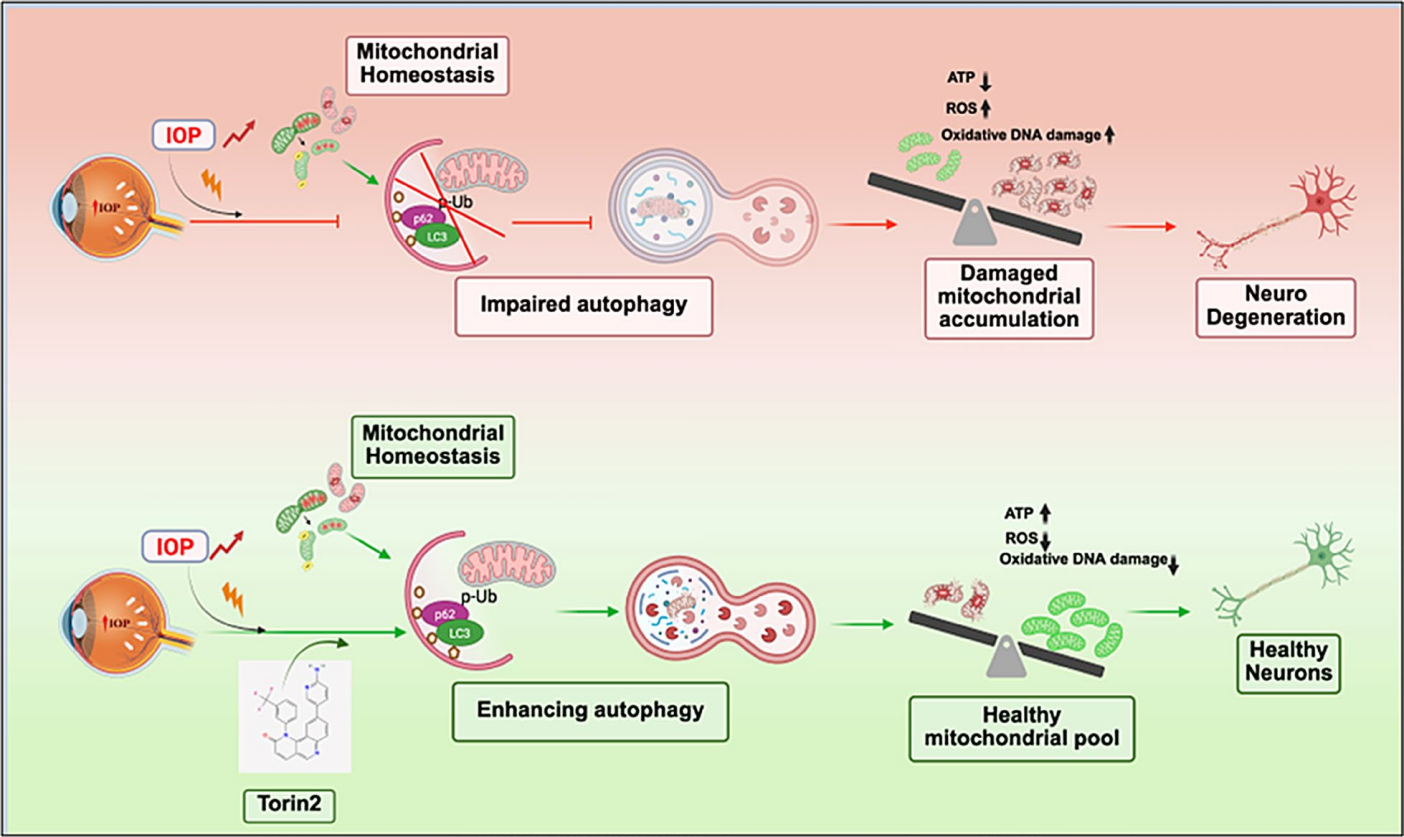

**Supplementary Information:**

The online version contains supplementary material available at 10.1186/s13024-026-00950-4.

## Introduction

Glaucoma is a multifactorial neurodegenerative disease characterized by progressive loss of retinal ganglion cells (RGCs) and degeneration of the optic nerve (ON) axons, leading to irreversible vision loss. Glaucoma affects nearly 80 million people worldwide and is estimated to reach 111.8 million by 2040, making it the second leading cause of blindness in the world [1–3]. Despite its high prevalence, the underlying pathological mechanisms responsible for glaucomatous neurodegeneration remain poorly defined. Elevated intraocular pressure (IOP) due to trabecular meshwork (TM) dysfunction is the major risk factor for the onset and progression of glaucoma [4, 5]. Chronic elevation of IOP induces pathological changes at the optic nerve head (ONH), leading to RGC degeneration [6, 7]. These changes are accompanied by impaired axonal transport, glial/astrocyte activation, increased metabolic stress, and neuroinflammation [6, 8–14]. The primary therapeutic approach to the management of glaucoma is focused on reducing IOP. However, vision loss continues to progress in some glaucoma patients despite adequate therapeutic control of IOP, suggesting that additional factors contribute to the progression of the disease [15–18]. Therefore, identifying and targeting IOP-induced early events of glaucomatous neurodegeneration is crucial for developing effective glaucoma treatments.

Degeneration of RGC somas and axons is the pathological hallmark of glaucoma [7, 19–21]. RGCs form the innermost layer of the neural retina and are the only retinal neurons communicating directly with the brain. RGCs receive vast visual information from the photoreceptors via retinal interneurons (including horizontal, bipolar, and amacrine cells) and relay visual information in the form of action potentials to the visual centers of the brain. Therefore, RGCs rely mainly on mitochondria to support their high bioenergetic requirements and maintain their complex structure and functional integrity [22]. In RGCs, mitochondria redistribute continuously across dendrites, axons, and synapses to meet high bioenergetic demands via fission and fusion events [23–26]. RGCs use approximately 90% of mitochondrial-generated ATP to maintain membrane dynamics essential to sustain action potentials and survival [27–30]. In addition to providing the majority of cellular ATP energy via oxidative phosphorylation (OXPHOS), mitochondria also play a vital role in RGCs by regulating Ca^2 +^ homeostasis, apoptotic and redox signaling, and promoting neurotransmission and synaptic plasticity [31, 32]. Thus, mitochondrial dysfunction in RGCs can profoundly affect neuronal function and survival. Emerging evidence of mitochondrial dysfunction has been identified in a wide variety of age-related and neurodegenerative diseases, including glaucoma [28, 29, 33–43]. A spectrum of mitochondrial abnormalities, including increased mitochondrial DNA (mtDNA) mutations and reduced mitochondrial respiratory functions, has been reported in patients with primary open-angle glaucoma (POAG), the most common form of glaucoma [44–47]. Additionally, exposure of cultured RGCs to elevated hydrostatic pressure has been shown to alter mitochondrial dynamics, resulting in reduced ATP production [39, 48]. Therefore, a deeper understanding of the mechanisms behind mitochondrial dysfunction and glaucomatous neurodegeneration is essential for providing new insights into neurodegeneration and advancing targeted treatments.

Mitochondria within RGCs are vulnerable due to their postmitotic state, high metabolic demands, increased oxidative stress, challenges in mitochondrial transport, and exposure to environmental and pathological stressors [29, 49–51]. As a result, the stressed or dysfunctional mitochondria within RGCs undergo constant repair or degradation. Consequently, the cellular processes regulating mitochondrial turnover are essential for the survival and proper functioning of RGCs [52–54]. Autophagy is an intracellular process that involves the sequestration of damaged cytoplasmic components, including organelles and protein aggregates, into double-membraned autophagosomes, followed by their degradation in lysosomes. Autophagic degradation of damaged mitochondria is termed mitophagy, a major mitochondrial quality control mechanism in neurons that allows selective degradation of damaged mitochondria [55–57]. This pathway is coordinated by autophagy-related (ATG) proteins, including the ATG5–ATG12–ATG16L1 complex, which is required for phagophore expansion, and the lipidation of LC3B. The cargo receptor p62/SQSTM1 recognizes polyubiquitinated substrates and links them to LC3B, facilitating selective autophagy [58–60]. Impairment of autophagy can result in the accumulation of dysfunctional mitochondria, contributing to cellular stress and degeneration [61, 62]. While autophagy has been studied in the context of glaucoma [54, 63–68], its direct impact on mitochondrial homeostasis in RGCs has not been adequately explored. This is a critical gap, as mitochondrial dysfunction is increasingly recognized as a central contributor to RGC vulnerability and degeneration in glaucoma [23, 28, 29, 35, 37–45, 48–50, 53, 69–79].

To further elucidate the role of autophagy and mitochondrial dysfunction in the etiology of glaucomatous neurodegeneration, we utilized two distinct mouse models of glaucoma: glucocorticoid (GC)-induced ocular hypertension (OHT) [80, 81] and a Cre-inducible mouse model of MYOC-associated POAG (*Tg.Cre-MYOC*^*Y437H*^) [82, 83]. Both mouse models closely mimic the clinical and morphological characteristics of human POAG, including IOP elevation due to TM dysfunction, leading to well-defined early events of glaucomatous neurodegeneration. Using these two mouse models of glaucoma, we demonstrate that IOP elevation leads to the accumulation of damaged mitochondria in RGCs and ON. Using Mt-Keima mitophagy reporter mice and RGC-specific *Atg5* conditional knock-out mice, we demonstrate that defective autophagy contributes to dysfunctional mitochondrial accumulation and glaucomatous neurodegeneration. Interestingly, enhancing autophagy using the pharmacological agent Torin 2 restored mitochondrial health and prevented glaucomatous neurodegeneration in both mouse model of glaucoma and ex vivo cultured human retinal explants. Our results demonstrate that impaired autophagy and mitochondrial turnover drive glaucomatous neurodegeneration, while enhancing autophagy restores mitochondrial function and promotes neuroprotection.

## Results

### Ocular hypertension leads to the accumulation of damaged mitochondria in RGCs and ON axons in mouse models of glaucoma

**Fig. 1:**
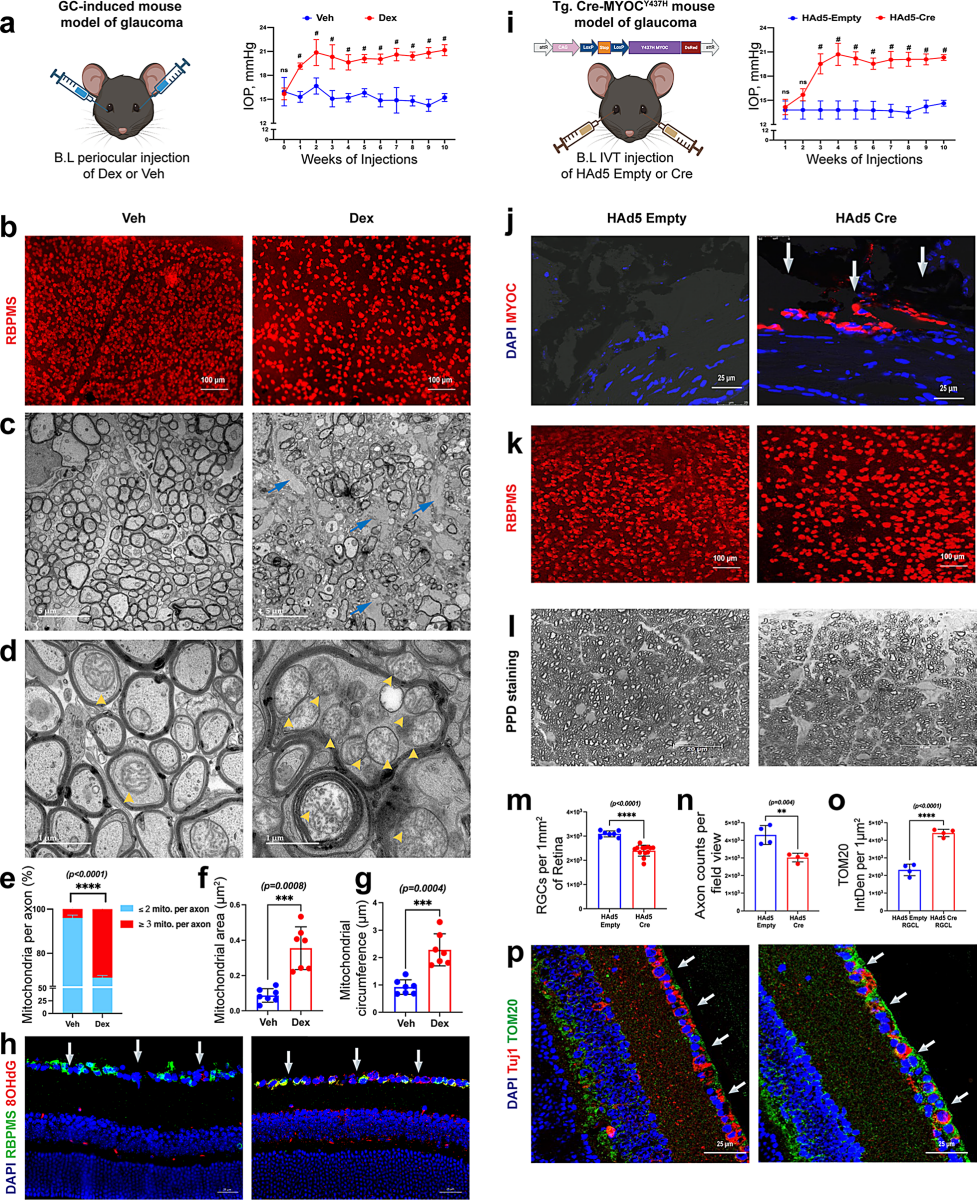
IOP elevation leads to the accumulation of damaged mitochondria in RGCs of mouse models of glaucoma. **a**) Three-month-old C57BL/6J mice received bilateral (B.L.) periocular injections of Veh or Dex (200 µg/eye) once a week for 10 weeks. Dex-injected mice showed significant IOP elevation starting from the first week of injections compared to Veh-injected mice (*n* = 10 in each group, #*p < 0.0001*, ns, not significant). **b**) Chronic elevation of IOP in Dex-injected mice resulted in a significant loss of RGCs (*n* = 5 in each group) based on whole-mount retinal immunostaining with the RGC-specific marker RBPMS. TEM imaging of ON cross-sections showed **c**) severe axonal degeneration in Dex-injected eyes compared to Veh-injected eyes (blue arrows represent glial scars), and **d**) the accumulation of mitochondria in Dex-induced OHT eyes compared to Veh-injected eyes (yellow arrowheads represent mitochondria within the axons). **e**) Stacked bar graphs representing the quantitative analysis of the percentage of mitochondria per axon. Quantitative analyses of the axonal mitochondrial **f**) area and **g**) circumference (*n* = 7 ONs from each group). **h**) Immunostaining data showed increased expression of 8-OHdG in the RGCL of Dex-induced OHT eyes compared to Veh-injected eyes (*n* = 4–5 per group; arrows represent RGCL). **i**) Three-month-old *Tg.Cre-MYOC*^*Y437H*^ mice were injected intravitreally with either HAd5-empty or Cre (2 × 10⁷ pfu/eye), and IOP was monitored weekly. A significant IOP elevation was observed in *Tg.Cre-MYOC*^*Y437H*^ mice injected with HAd5-Cre compared to HAd5-empty-injected mice (*n* = 4–5 in each group, ns, not significant, *#p < 0.0001*). **j**) Imaging of anterior segment cross-sections showed the accumulation of DsRed-tagged mutant myocilin specifically in the TM region (arrows) of HAd5-Cre-injected *Tg.Cre-MYOC*^*Y437H*^ mice compared to HAd5-empty-injected mice. Chronic IOP elevation in *Tg.Cre-MYOC*^*Y437H*^ mice resulted in **k **&** m**) a significant loss of RGCs (~ 22% loss, *n* = 7–13 in each group); **l **&** n**) ~ 30% axonal degeneration in PPD-stained ON (*n* = 4 in each group); and **o & p**) increased expression of TOM20 in the RGCL of HAd5-Cre-injected *Tg.Cre-MYOC*^*Y437H*^ mice compared to HAd5-empty-injected mice (*n* = 4 in each group)

To investigate whether mitochondrial dysfunction is associated with glaucomatous neurodegeneration, we have utilized a mouse model of GC-induced glaucoma and *Tg.Cre-MYOC*^*Y437H*^ mice, which mimic the pathophysiology of POAG [80, 82, 83]. GC-induced OHT was achieved by administering dexamethasone (Dex) via the periocular route (200 µg/eye) once weekly for 10 weeks, as described previously [80, 81, 84, 85]. Dex-injected eyes showed significant IOP elevation compared to the Veh-injected control eyes (Fig. 1a). Consistent with our previous study [80], Dex-induced IOP elevation for 10 weeks resulted in significant loss of RGCs (Fig. 1b, Fig. S1a) and ON axonal degeneration (Fig. 1c, Fig. S1 b). We utilized this mouse model to evaluate whether Dex-induced OHT leads to mitochondrial damage in RGCs. Transmission Electronic Microscopy (TEM) on cross sections of ON revealed that glaucomatous neurodegeneration is associated with thinning of the myelin sheath, glial scar formation, and accumulation/an increased number of mitochondria within the degenerating axons of Dex-induced OHT eyes compared to the Veh-injected eyes (Fig. 1c). Notably, higher-magnification TEM images revealed that the accumulated mitochondria in the ON of Dex-induced OHT eyes appeared damaged, as evidenced by their swollen morphology and disorganized or fragmented cristae (Fig. 1d). Additionally, there was a significant increase in the number of mitochondria per axon (Fig. 1e, Fig S1c & d), along with increased mitochondrial area (Fig. 1f, Fig S1c) and circumference (Fig. 1g, Fig S1c). We further confirmed these findings by immunostaining mitochondrial marker TOM20 and oxidative DNA damage indicator 8-Hydroxy-2’-deoxyguanosine (8-OHdG) (which detects nuclear and mitochondrial DNA damage) on retinal cross-sections. Consistent with TEM analysis, we observed increased 8-OHdG staining (Fig. 1h, Fig S1e) and TOM20 (Fig. S1f, g & h) in the RGC layer (RGCL) of Dex-induced OHT retinas compared to the Veh-injected control retinas. These data indicate that chronic IOP elevation leads to the accumulation of structurally aberrant mitochondria in RGCs and oxidative DNA damage to RGCs in a mouse model of Dex-induced glaucoma.

Next, we explored whether the accumulation of mitochondria is also associated with glaucomatous neurodegeneration in our recently developed Cre-inducible mouse model of POAG expressing a DsRed-tagged Y437H mutant of human myocilin (*Tg.Cre-MYOC*^*Y437H*^). Upon introduction of Cre, the STOP cassette, which inhibits transgene expression, is excised, and mutant MYOC-DsRed is explicitly expressed in the TM tissue, elevating IOP in *Tg.Cre-MYOC*^*Y437H*^ mice [82, 83]. Adult *Tg.Cre-MYOC*^*Y437H*^ mice were injected intravitreally with helper adenovirus (HAd) 5 expressing empty cassette or Cre under CMV promoter (2 × 10^7^ pfu/eye). The expression of mutant MYOC in the TM resulted in sustained and significant IOP elevation, which started at 2 weeks of injection (Fig. 1i & j). Chronic IOP elevation for 10 weeks led to glaucomatous neurodegeneration, including significant loss of RGC and ON axonal degeneration (Fig. 1k-n). Similar to the GC-induced mouse model of glaucoma, glaucomatous neurodegeneration in *Tg.Cre-MYOC*^*Y437H*^ mouse model was also associated with the accumulation of mitochondria in RGCL (Fig. 1o, p) and degenerative axons (Fig. S1i). These data indicate that the accumulation of swollen or structurally abnormal mitochondria and oxidative stress occurs in RGC soma and axons during the early stage of glaucomatous neurodegeneration.

### Ocular hypertension impairs autophagy in RGCs, leading to the accumulation of damaged mitochondria prior to glaucomatous neurodegeneration

**Fig. 2:**
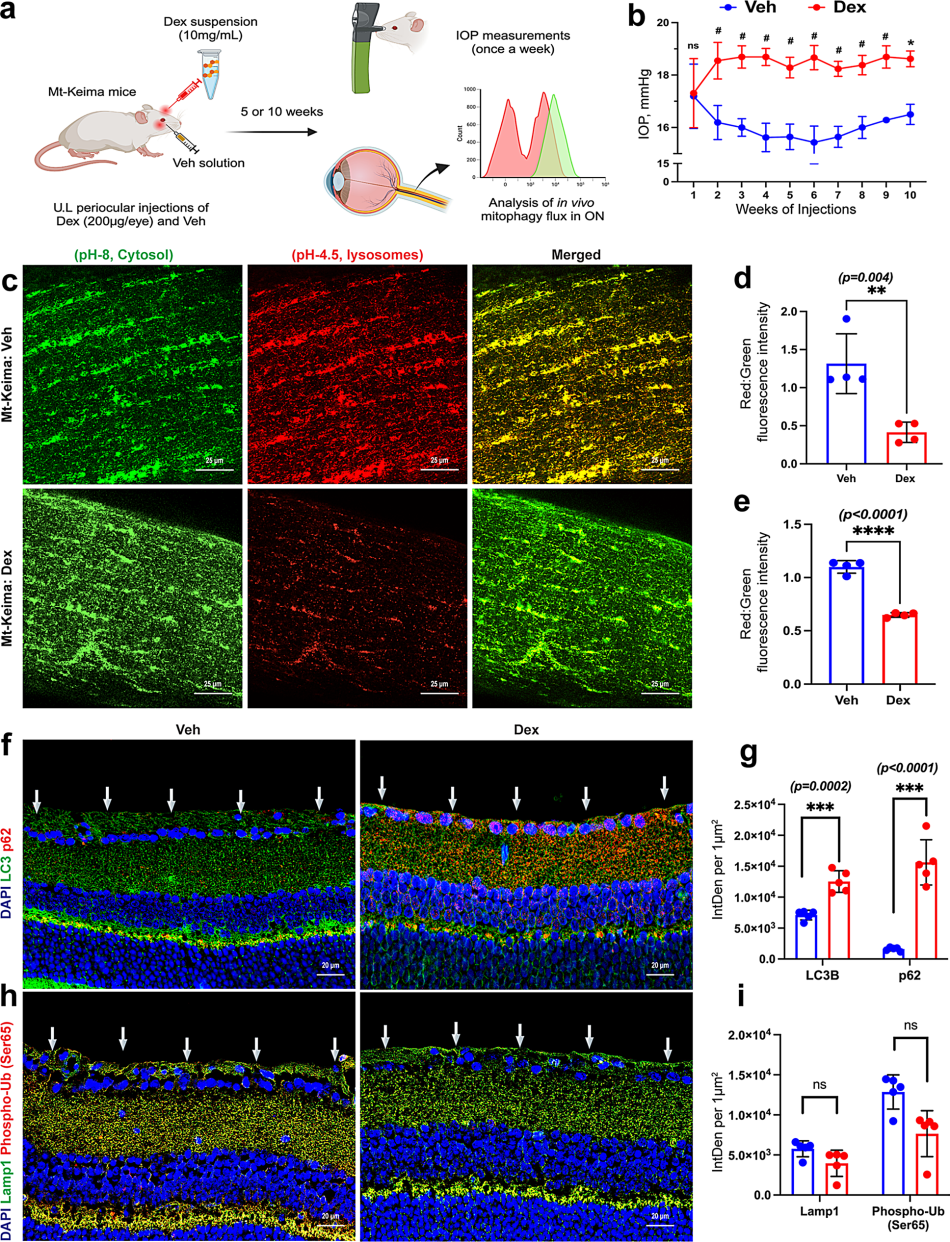
Autophagy/mitophagy impairment precedes glaucomatous neurodegeneration. **a**) A schematic illustration of the study design. Mt-Keima reporter mice were injected unilaterally (U.L) with either Dex (200 µg/eye) or Veh once a week for 5 or 10 weeks via the periocular route. IOP was recorded weekly. Following 5 and 10 weeks of OHT, mitophagy flux was measured in the ON of Mt-Keima mice treated with Veh or Dex. b) Dex-treated Mt-Keima mice demonstrated sustained and significant IOP elevation compared to contralateral Veh-injected eyes (n = 8 in each group, #p < 0.0001, ns-not significant). **c**) Representative images showing the red (excitation 561 nm) and green (excitation 458 nm) fluorescence intensities in Mt-Keima mouse ON after 5 weeks of Dex-induced OHT. Quantitative analysis of red: green fluorescence showed a significant reduction in mitophagy flux in both **d**) 5-week and **e**) 10-week Dex-injected Mt-Keima mouse ON compared to contralateral Veh-injected ON (n = 4 in each group). **f**) Immunostaining and g) quantification of integrated fluorescence density showed increased LC3B and p62 expression in the RGCL of Dex-injected mice (C57BL/6J) compared to Veh after 5 weeks of OHT (n = 5 in each group; arrows represent RGCL). **h** & **i**) Expression of Lamp1 and phospho-Ub (Ser65) in the RGCL of Dex-injected mice (C57BL/6J) compared to Veh after 5 weeks of OHT (n = 5 in each group; arrows represent RGCL)

We hypothesize that sustained OHT impairs mitophagy, resulting in the abnormal accumulation of damaged mitochondria preceding neurodegeneration. To test this, we utilized the Mt-Keima reporter transgenic mouse model, which enables the ex vivo assessment of mitophagy flux. Mt-Keima mice express a mitochondria-tagged fluorescent protein, Keima, which is both pH-sensitive and resistant to lysosomal proteases [86]. The Mt-Keima protein appears green in a neutral pH environment (cytosol) and red in an acidic environment (lysosomes), allowing quantification of mitophagy flux as mitochondria transition from autophagosomes to lysosomes. As illustrated in the schematic (Fig. 2a), Mt-Keima mice received periocular injections of Dex (200 µg) in one eye, while the contralateral eye received Veh once weekly for either 5 or 10 weeks. Dex-treated eyes showed a significant IOP elevation compared to the contralateral Veh-injected eyes (Fig. 2b). To assess mitophagy dynamics at different stages of disease progression, ex vivo mitophagy flux was examined in the whole ON at both 5 weeks (prior to axonal degeneration) and 10 weeks (during axonal degeneration) using confocal microscopy with excitation at 458 nm (green) and 561 nm (red). Consistent with our previous findings [80], Dex-induced OHT for 5 weeks did not cause RGC loss (Fig. S2a & b) and was defined as the pre-neurodegeneration stage, while the 10-week time point represented the neurodegenerative stage (Fig. 1b, c, Fig.S1a, b). Mitophagy flux was measured at both time points to determine whether mitophagy impairment occurs as an early, potentially causative event preceding neurodegeneration, or as a consequence of ongoing axonal damage. Representative images of the whole ON (Fig. 2c) and their quantitative analyses (Fig. 2d) demonstrated a reduced ratio of red fluorescence (mitochondria in lysosomes) over green fluorescence (mitochondria in the cytoplasm) after 5 weeks of Dex-induced OHT, indicating that mitophagy flux is significantly reduced prior to neurodegeneration. Although not statistically significant, a similar trend was observed in the wholemount retina, with a decreased red-to-green fluorescence ratio at 5 weeks (Fig. S2 c & d). Significantly reduced mitophagy flux persisted at 10 weeks of OHT (Fig. 2e), indicating that mitophagy impairment is sustained during neurodegeneration. These data suggest that mitophagy impairment precedes and potentially contributes to glaucomatous neurodegeneration.

To further support these findings, we examined the expression of autophagy-related markers p62 (SQSTM1) and LC3B in mouse models of glaucoma prior to axonal degeneration. Increased levels of p62 and LC3B proteins were observed in RGCs of both GC-induced (Fig. 2f & g) and *Tg.Cre-MYOC*^*Y437H*^ (Fig. S2e-h) mouse models of glaucoma, consistent with impaired autophagy. These results suggest that defective autophagy/mitophagy contributes to the accumulation of damaged mitochondria and promotes glaucomatous neurodegeneration. To further determine whether impaired autophagy/mitophagy is associated with glaucomatous neurodegeneration, we assessed the expression of phosphorylated ubiquitin at serine 65 (phospho-Ub (Ser65)), a hallmark of damaged mitochondria tagged for PTEN-induced kinase (PINK) 1/Parkin-mediated mitophagy (Fig. 2h & i). In neurons, PINK1/Parkin-mediated mitophagy is one of the most extensively studied mechanisms for eliminating damaged mitochondria [50, 87–89]. Phospho-Ub (Ser65) is generated when PINK1 accumulates on the outer mitochondrial membrane of depolarized mitochondria, where it phosphorylates ubiquitin to recruit and activate Parkin, thereby initiating the autophagy cascade [90–94]. In the GC-induced glaucoma model, phospho-Ub staining in RGCs showed a downward trend (Fig. 2h & i), and this modest decline may indicate insufficient activation of the PINK1/Parkin pathway, further supporting the notion of impaired mitochondrial turnover in glaucomatous retina. Importantly, LAMP1 expression remained unaltered (Fig. 2h & i), indicating that lysosomal integrity is preserved and that the disruption is likely specific to autophagic flux. These data indicate that impaired autophagy and mitophagy precede and contribute to the accumulation of damaged mitochondria and the progression of glaucomatous neurodegeneration.

### Deficient autophagy in RGCs leads to the accumulation of damaged mitochondria and subsequent neurodegeneration

**Fig. 3:**
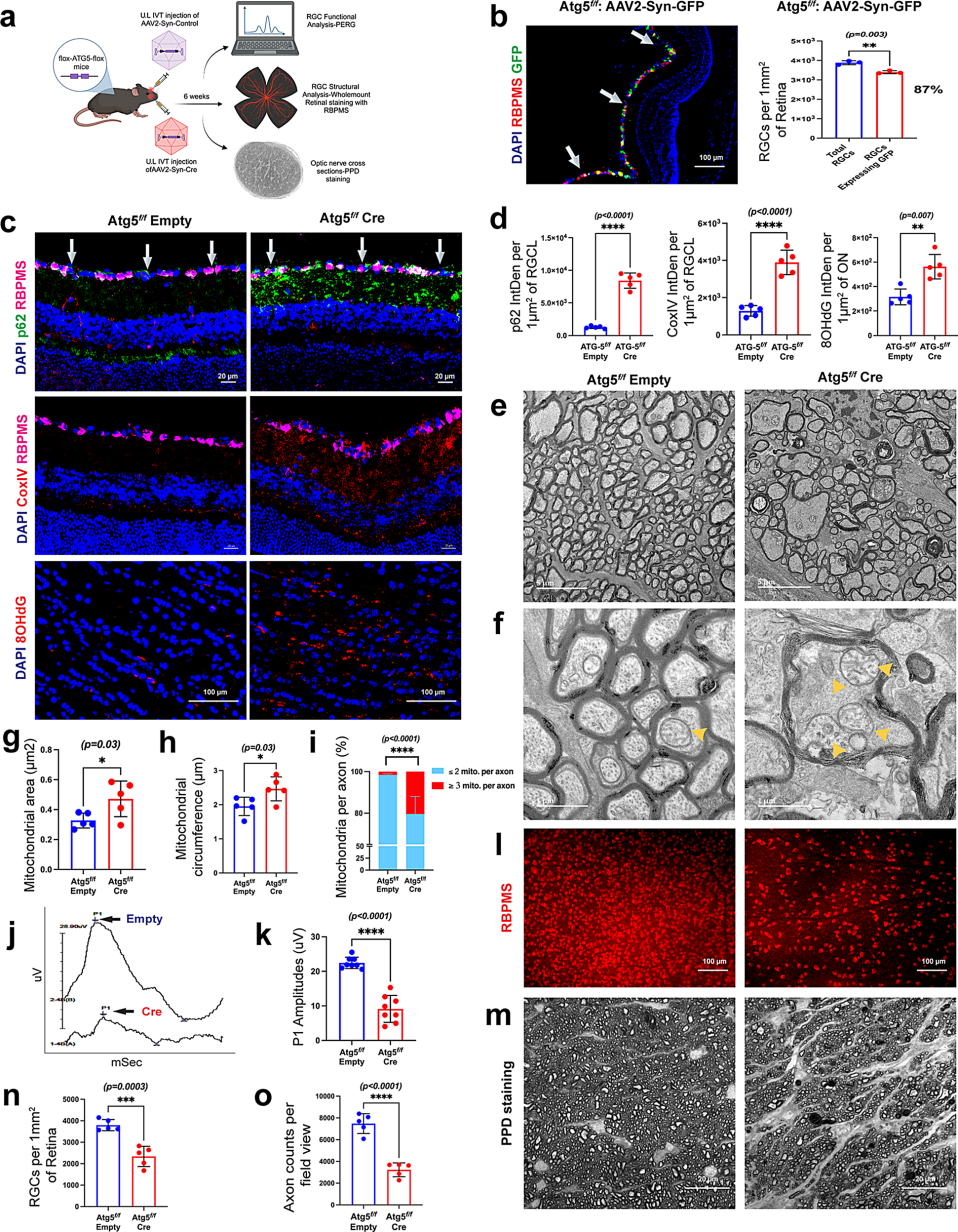
RGC-specific conditional knockdown of *Atg5* induces the accumulation of damaged mitochondria, leading to neurodegeneration.** a**) A schematic illustration of the study design. Three-month-old *Atg5*^*flox/flox*^ mice were intravitreally injected in one eye with AAV2-Syn-Cre and the contralateral eye with an AAV2-Syn-empty vector. Mitochondrial accumulation and neurodegeneration were evaluated 6 weeks after injection. **b**) AAV2-Syn-GFP was injected intravitreally, and GFP expression in RGCs was examined in retinal cross-sections (left panel), showing 87% transduction efficiency based on whole-mount retinal counting. Co-labeling of GFP with RBPMS demonstrated the specific tropism of AAV2-Syn-GFP for RGCs. **c **&** d**) Representative immunostaining images and their intensity measurements demonstrate significantly increased levels of p62 and COXIV in the RGCL, along with 8-OHdG in the ON, in AAV2-Syn-Cre-injected eyes compared to contralateral AAV2-Syn-empty vector-injected eyes of *Atg5*^*flox/flox*^ mice (arrows represent RGCL; *n* = 5 in each group). **e **&** f**) Representative TEM images show accumulation of damaged mitochondria in AAV2-Syn-Cre-injected eyes compared to empty vector-injected eyes of *Atg5*^*flox/flox*^ mice (yellow arrowheads represent mitochondria within ON axons); *n* = 5 in each group. Quantitative analysis of axonal mitochondria revealed differences in (**g**) area, (**h**) circumference, and (**i**) mitochondrial percentage per axon. RGC-specific conditional knockdown of *Atg5* resulted in (**j **&** k**) decreased RGC function, as evident from reduced PERG amplitudes (21.78 µV vs. 8.61 µV) (*n* = 8 in each group), (**l **&** n**) significant RGC loss, as determined by whole-mount retinal staining with RBPMS (~ 39% loss) (*n* = 5 in each group), and (**m **&** o**) significant loss of healthy axons (~ 59%), as determined by PPD staining 6 weeks after AAV2-Syn-Cre injection (*n* = 5 in each group)

Since autophagy machinery is required for functional mitophagy, we sought to determine whether conditional loss of autophagy selectively in RGCs leads to the accumulation of dysfunctional mitochondria and subsequent neurodegeneration using *Atg5*^*flox/flox*^ mice. The *Atg5* gene encodes the autophagy-related protein ATG5, which plays a critical role in autophagy as part of the ATG12–ATG5–ATG16L1 complex. ATG5 is essential for the elongation of the phagophore membrane and is required for the conjugation of LC3 to phosphatidylethanolamine (PE), a key step in autophagosome formation. It is indispensable for canonical autophagy and also contributes to various forms of selective autophagy, including PINK1/Parkin-mediated mitophagy, facilitating the sequestration, and degradation of damaged mitochondria via the lysosomal degradation pathway [95–99]. To investigate the role of basal autophagy in RGCs, we performed a conditional knockdown of *Atg5* by intravitreal injection of an adeno-associated viral vector 2 (AAV2) expressing Cre recombinase under the control of the synapsin promoter (AAV2-Syn-Cre) into *Atg5*^*flox/flox*^ mice, thereby enabling RGC-specific disruption of autophagy and mitophagy. As illustrated in the schematic (Fig. 3a), three-month-old *Atg5*^*flox/flox*^ mice received intravitreal injections of AAV2-Syn-Cre (1.67E + 13 VG/mL) in one eye, while the contralateral eyes were injected with AAV2-Syn-Empty as a control. Structural and functional loss of RGCs and axonal degeneration were examined 6 weeks after the injection. To determine whether AAV-Syn exhibits specific tropism to RGCs, C57BL/J mice were injected with AAV-Syn-GFP. Retinal cross-sections (left panel) and whole mount retina (right panel) staining with RGC marker, RBPMS, revealed selective GFP expression in RBPMS-positive RGCs with 87% transduction efficiency, indicating that AAV-Syn exhibits specific tropism to RGCs (Fig. 3b). Moreover, we further determined whether AAV2-Syn-Cre induces any toxicity. C57BL/6J mice injected with AAV2-Syn-Cre showed no evidence of Cre-mediated toxicity compared with the contralateral eyes injected with empty vector control, as assessed by Annexin V staining (Fig. S3a) and pERG analysis (Fig. S3b &c). Together, these findings validate AAV-Syn-Cre as a safe and effective approach for RGC-targeted gene manipulation in vivo. Loss of RGC-specific *Atg*5 resulted in the accumulation of p62 and COXIV in RGCL with increased 8-OHdG levels in ON (Fig. 3c & d). Furthermore, TEM images confirmed the accumulation of damaged mitochondria in the ON of *Atg5* knockout eyes (Fig. 3e & f), characterized by increased mitochondrial area (Fig. 3g, Fig. S3d), enlarged circumference (Fig. 3h, Fig. S3d), and a higher number of mitochondria per axon (Fig. 3i, Fig. S3e), consistent with impaired clearance due to loss of autophagy. These data indicate that impaired autophagy due to RGC-specific loss of *Atg5* leads to the accumulation of damaged mitochondria and oxidative DNA damage in RGCs. Next, we examined whether loss of autophagy in RGCs is sufficient to induce neurodegeneration in mice. pERG analysis demonstrated a significant functional loss of RGCs in *Atg5*^*flox/flox*^ mice eyes injected with AAV2-Syn-Cre (Fig. 3j & k). The knockdown of RGC-specific *Atg*5 resulted in a significant loss of RGC (~ 39%) (Fig. 3i & n) and severe axonal loss (~ 59%) after 6 weeks of injection (Fig. 3m & o). Together, these data indicate that loss of RGC-specific autophagy leads to abnormal accumulation of damaged mitochondria in RGCs, leading to oxidative DNA damage and neurodegeneration.

### Enhancement of autophagy via Torin 2 prevents glaucomatous neurodegeneration in mouse models of glaucoma and ex vivo human retinal explant model

**Fig. 4:**
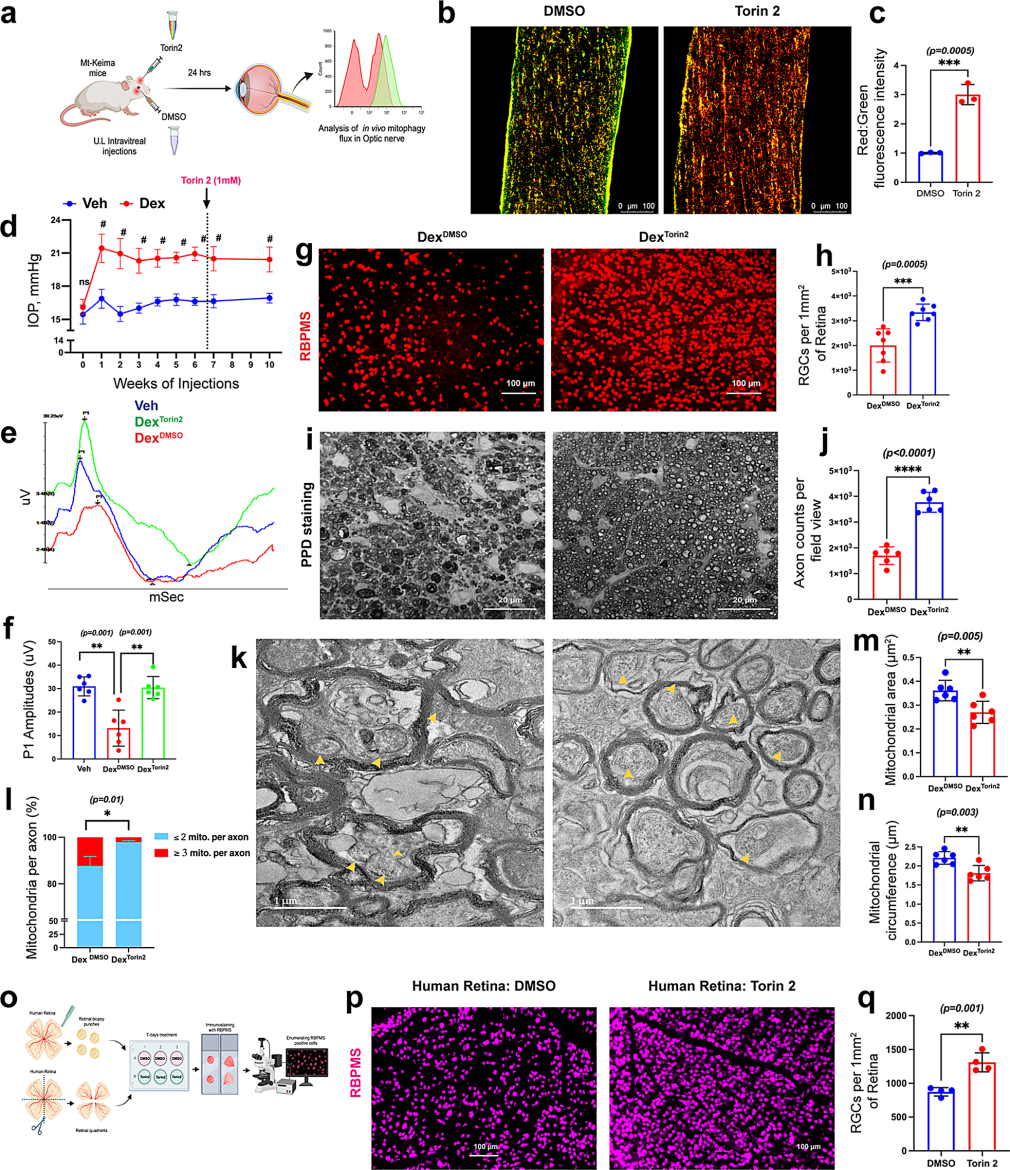
Enhancing autophagy via Torin 2 prevents damaged mitochondrial accumulation in RGCs and mitigates glaucomatous neurodegeneration.** a**) A schematic illustration of the study design. Mt-Keima mice were intravitreally injected with Torin 2 (1 mM) in one eye and DMSO in the contralateral eye. Mitophagy flux was examined 24 h after treatment. **b & c**) Representative images and their fluorescence intensity analysis showed a significant increase in the red-to-green ratio (mitophagy flux) in the Torin 2-treated ON compared to the contralateral DMSO-treated ON of Mt-Keima mice (*n* = 3 in each group). **d**) Ocular hypertensive C57BL/6J mice were treated intravitreally with Torin 2 (1 mM) in one eye, and the contralateral eyes were injected with DMSO as control. A sustained elevation of IOP was maintained for another three weeks by administering Dex periocular injections. After 10 weeks of injections, glaucomatous neurodegeneration was assessed. Dex-injected mice exhibited a significant and sustained increase in IOP throughout the study (#*p < 0.0001*; *n* = 8–10 eyes in each group). **e & f**) Representative PERG waves and statistical analysis showed that Torin 2 significantly improved PERG amplitudes in Dex-treated eyes compared to Dex and DMSO-treated eyes (*n* = 6 in each group). **g & h**) Representative whole-mount retinal staining with RBPMS (*n* = 6–7 in each group) and **i & j**) PPD-stained ON axons (*n* = 6 in each group) showed an increased number of RGCs and healthy axons in Dex-induced OHT eyes treated with Torin 2 compared to contralateral Dex-induced OHT eyes treated with DMSO, respectively. **k**) Representative TEM images show reduced accumulation of damaged mitochondria in Torin 2-treated eyes compared to DMSO-treated eyes (yellow arrowheads represent mitochondria within ON axons); *n* = 6 in each group. Quantitative analyses of axonal mitochondria showed changes in (**l**) percentage per axon, (**m**) area, and (**n**) circumference. **o**) A study design illustrating Torin 2 treatment in human retinal explants. Retinal explants were cultured for 7 days under neurotrophic factor deprivation conditions and treated with DMSO or Torin 2 (100 µM) in the explant medium. Surviving RGCs were quantified using an anti-RBPMS antibody. **p & q**) Torin 2-reated retinal explants showed a higher number of RGCs than those treated with DMSO (*n* = 4 in each group)

Since our findings suggest impaired autophagy contributes to glaucomatous neurodegeneration, we next examined whether enhancing autophagy in RGCs could mitigate disease progression. We evaluated whether Torin 2, a potent and selective mTOR inhibitor that activates autophagy, could enhance autophagy flux and mitigate glaucomatous pathology. First, we investigated whether Torin 2 enhances mitophagy flux using Mt-Keima mice. 3-month-old Mt-Keima mice were intravitreally injected with Torin 2 (1 mM dissolved in DMSO) in one eye, and the contralateral eyes were injected with DMSO as a control (Fig. 4a). Ex vivo mitophagy flux measurements demonstrated a significant enhancement of mitophagy flux 24 h after Torin 2 treatment (Fig. 4b, c). Next, we examined whether Torin 2 prevents neurodegeneration in a mouse model of GC-induced glaucoma. 3-month-old C57BL/6J mice were treated with Dex once a week for 7 weeks. IOPs were monitored to ensure a significant elevation in IOP. At 7 weeks of OHT, Dex-treated mice were injected intravitreally with Torin 2 (1 mM dissolved in DMSO) in one eye, and the contralateral eyes were injected with DMSO as a control. Dex-treatment was continued for another 3 weeks to maintain OHT (Fig. 4d). IOP measurements showed that Dex-treated eyes continued to have significant IOP elevation, and Torin 2 did not alter IOPs in Veh and Dex-treated eyes (Fig. 4d). At the end of the 10-week study period, pERG measurements demonstrated that Torin 2 treatment significantly preserved RGC function in Dex-treated eyes (Dex^Torin2^), with mean amplitudes of 31.82 µV, comparable to non-OHT vehicle-treated eyes (33.04 µV) while Dex^DMSO^ control eyes showed significantly reduced amplitudes (17.36 µV) (Fig. 4e & f). Consistent with enhanced RGC function, Torin 2 treatment significantly preserved RGCs survival, with a 37% increase in RGCs number (Fig. 4g & h) and a 51% increase in the proportion of morphologically intact axons in Dex^Torin2^ treated eyes compared to Dex^DMSO^ controls eyes (Fig. 4i & j). Additionally, enhancement of autophagy through Torin 2 treatment improved the healthy mitochondrial pool in RGCs and reduced the accumulation of damaged mitochondria in the Dex-induced mouse model of glaucoma (Fig. 4k-n, Fig. S4a & b). Consistently, Torin 2 treatment preserved anterograde axonal transport, as evidenced by robust CTB transport to the superior colliculus (SC), indicating improved axonal integrity and function (Fig. S4c). Together, these data suggest that the enhancement of autophagy via Torin 2 prevents glaucomatous neurodegeneration in a mouse model of Dex-induced glaucoma.

Next, we also examined whether Torin 2 prevents neurodegeneration in the recently developed *Tg.Cre-MYOC*^*Y437H*^ mouse model of glaucoma [100]. Three-month-old *Tg.Cre-MYOC*^*Y437H*^ mice were intravitreally injected with HAd5-Cre (2 × 10⁷ pfu/eye) to induce mutant myocilin expression selectively in the TM, while control mice received HAd5-empty. IOPs were monitored weekly to confirm sustained elevation. After 7 weeks of OHT, mice received intravitreal Torin 2 (1 mM dissolved in DMSO) in one eye, while the contralateral eye received DMSO as control. IOP measurements showed that Torin 2 treatment significantly reduced IOP in HAd5-Cre–injected *Tg.Cre-MYOC*^*Y437H*^ mice (Fig. S4d). At the end of the 10-week experimental period, Torin 2 treatment significantly prevented RGC loss (Fig. S4e & f). Consistent with improved RGC numbers, pERG recordings demonstrated significant preservation of RGC function in Torin 2-treated eyes compared with DMSO-treated control eyes (Fig. S4g & h). Together, these results indicate that enhancing autophagy with Torin 2 protects RGCs and preserves visual function in the *Tg.Cre-MYOC*^*Y437H*^ mouse model of glaucoma.

We further confirmed these findings using an ex vivo human retinal explant model [101, 102]. To first determine whether Torin 2 effectively activates autophagy in retinal tissue, retinal explants were treated with either DMSO or Torin 2 (100 µM) for 12 h, and western blot analysis showed reduced p62 expression compared with DMSO controls, indicating enhanced autophagic activity (Fig. S4i & j). Next, to evaluate the neuroprotective effect of Torin 2, retinal explants were treated with either DMSO or Torin 2 (100 µM) and cultured for 7 days in explant medium under neurotrophic factor-deprived conditions. The explants were then fixed, and surviving RGCs were quantified using an anti-RBPMS antibody (Fig. 4o). Torin 2 treatment significantly increased the total number of surviving RGCs compared with DMSO-treated controls (Fig. 4p, q), resulting in approximately a 49.7% increase in RGC survival.

Together, these findings highlight the neuroprotective role of autophagy, indicating that pharmacological enhancement of autophagy improves mitochondrial turnover and attenuates glaucomatous neurodegeneration.

## Discussion

Although mitochondrial dysfunction is a well-established feature of glaucomatous neurodegeneration, the mechanistic basis for the progressive accumulation of dysfunctional mitochondria in RGCs remains inadequately defined. In this study, we demonstrate that autophagy is significantly impaired in RGCs of mouse models of glaucoma. Elevated IOP disrupts autophagy, resulting in the accumulation of damaged mitochondria in RGC soma and axons. This mitochondrial pathology is accompanied by increased DNA oxidative damage, ultimately contributing to RGC degeneration. Consistent with these findings, we show that basal autophagy is essential for maintaining mitochondrial integrity and RGC survival. Notably, autophagy impairment and mitochondrial damage occur prior to neurodegeneration, suggesting that mitochondrial dysfunction due to its impaired turnover contributes to neurodegeneration. Importantly, pharmacological activation of autophagy via Torin 2 mitigates glaucomatous neurodegeneration in mouse models and ex vivo human retinal explants, highlighting autophagy as a potential therapeutic target for neuroprotection in glaucoma.

Several independent laboratories have demonstrated that altered mitochondrial dynamics and metabolic stress due to mitochondrial dysfunction are associated with glaucomatous neurodegeneration [53, 72, 103–106]. Previous studies by Drs. Simon John and Pete Williams, using the DBA/2J mouse model of inherited glaucoma, have shown that therapeutic strategies aimed at preserving mitochondrial function can significantly delay disease progression [53]. Notably, nicotinamide supplementation, a precursor of NAD⁺, has been shown to preserve mitochondrial integrity and protect against RGC loss in mouse models [53, 107–111], and combined nicotinamide and pyruvate supplementation improved visual function in patients with open-angle glaucoma [112]. Together, these studies highlight the crucial role of mitochondrial and metabolic resilience in protecting against glaucomatous damage.

Although mitochondrial dysfunction is a well-established contributor to glaucoma pathogenesis, the underlying molecular mechanisms contributing to abnormal mitochondrial accumulation in RGCs remain poorly understood. Autophagy, a highly conserved cellular process, plays a pivotal role in mitochondrial quality control by sequestering defective mitochondria into autophagosomes for subsequent lysosomal degradation, maintaining mitochondrial and metabolic balance, energy supply, neuronal survival, and health [69, 113]. Conversely, impaired autophagy promotes the accumulation of damaged mitochondria, leading to cellular dysfunction and neurodegeneration. There is mounting evidence from in vitro and in vivo studies that mitophagy/autophagy has neuroprotective effects in ocular neurodegenerative diseases, including glaucoma [54, 114–116]. Studies from Dr. Patricia Boya’s group have demonstrated that disruptions in mitophagy contribute to retinal and ON degeneration in age-related macular degeneration (AMD) [117–119], and impairment in this pathway has been linked to heightened vulnerability to oxidative stress and inflammation in the aging retina [120–122]. Additionally, Ambra1 haploinsufficiency has been shown to impair autophagy and accelerate retinal aging, highlighting the critical role of autophagy/mitophagy in maintaining retinal homeostasis and neuronal integrity [64, 123], and emphasizing the central role of mitochondrial quality control in retinal neuroprotection. Importantly, studies using human stem cell-derived RGCs [124, 125], and genetic studies using a large cohort of patients have identified mutations in mitophagy-related genes, such as TNK-binding kinase 1 (TBK1) and OPTN, associated with neurodegeneration [40, 71, 126–128]. Using Morrison’s model of OHT, we have recently demonstrated changes in mitophagosome formation that predispose RGCs to neurodegeneration in rats [129]. While many studies support a protective role for autophagy in RGC survival, some reports suggest that excessive or dysregulated activation of these pathways may contribute to RGC loss under glaucomatous stress conditions [65, 67, 68, 130, 131], highlighting a complex, context-dependent role for autophagy in glaucomatous progression. This duality underscores the need for further studies to better understand mitophagy and autophagy as potential therapeutic strategies for glaucoma. In this study, we used two well-established mouse models of glaucoma, including GC and mutant MYOC-induced OHT, which replicate key features of human glaucomatous neurodegeneration, making them appropriate models for studying disease mechanisms [80, 83]. These models feature well-defined timelines of neurodegeneration, allowing precise analysis of the onset and progression of RGC pathology. Importantly, the involvement of impaired autophagy was further corroborated by experiments in Mt-Keima mitophagy-reporter mice and RGC-specific *Atg5*^*flox/flox*^ conditional knock out mice.

PINK1-Parkin-mediated mitophagy is a significant and well-studied pathway in neurons, including RGCs [132–134]. To assess mitophagy ex vivo, we utilized Mt-Keima reporter mice, which express a pH-sensitive fluorescent protein targeted to mitochondria. This system enables robust and direct detection of mitochondria within the acidic environment of lysosomes, providing a reliable and quantifiable readout of mitophagic flux. The Mt-Keima model provides a precise ratiometric measurement of mitochondrial degradation, offering a distinct advantage for quantitative analysis of mitophagy. Notably, Mt-Keima mice have been reported to detect PINK1-Parkin-mediated mitophagy with higher sensitivity than other available reporter models [135], making them particularly well-suited for analyzing RGC-specific mitophagy under glaucomatous stress. While the mito-QC model, which relies on the differential stability of GFP and mCherry signals [136], has been instrumental in visualizing mitochondrial dynamics, Mt-Keima offers enhanced sensitivity and specificity for assessing mitophagy in vivo/ex vivo. Using Mt-Keima mitophagy reporter mice, we examined the effect of chronic IOP elevation on mitophagy flux both prior to axonal degeneration (5 weeks of OHT) and during axonal degeneration (10 weeks of OHT). Our previous studies demonstrated that axonal transport remains largely intact during the early stages of ON degeneration, whereas significant transport deficits emerge during more advanced stages of axonal injury in a GC-induced mouse model of glaucoma [80]. Interestingly, we observed a reduction in autophagy flux prior to the onset of axonal transport failure and structural degeneration of RGCs, suggesting that impaired autophagy is an early event in glaucomatous neurodegeneration. Given that autophagy is the primary mechanism for the clearance of damaged mitochondria, early disruption of this pathway may contribute to the accumulation of damaged mitochondria and increased susceptibility to subsequent injury. Furthermore, chronic OHT may exacerbate this process by impairing axonal transport, thereby limiting the delivery of mitochondria to lysosomes and compounding mitophagy deficits. This ultimately leads to a decrease in mitochondrial energy production and an increase in oxidative stress, which further exacerbate neuronal damage. Some studies have demonstrated that damaged mitochondria from distal axons are transported retrogradely to the neuronal soma for lysosomal degradation [137–140]. However, other studies have also found that dysfunctional mitochondria are degraded locally in distal axons via mitophagy rather than returning to the soma to prevent the spread of oxidative damage [141–144]. Nicholas Marsh-Armstrong et al. have also identified the existence of transcellular mitophagy at the ONH, where large numbers of mitochondria are shed from RGCs and degraded by the lysosomes of adjoining glial cells [142, 145]. Furthermore, the vulnerable or stressed axonal mitochondria exhibit reduced mitochondrial motility, preventing their return to the soma for degradation [141, 146]. Our findings demonstrate that autophagy impairment precedes axonal transport failure in glaucoma, highlighting early, local autophagy deficits as a potential driver of damaged mitochondrial accumulation and neurodegeneration.

Additionally, TEM imaging further confirmed the presence of these accumulated damaged mitochondria, which exhibited a swollen matrix (increased area and circumference) and disorganized cristae. Consistent with this, we observed elevated levels of 8-OHdG, a marker of oxidative DNA damage (including both nuclear and mitochondrial DNA), in RGCs, indicating ongoing chronic oxidative stress. Furthermore, we observed significantly increased levels of LC3b (detecting both the LC3-I and LC3-II forms) and p62, along with reduced phospho-Ub (Ser65) and unaltered LAMP1 expression prior to RGC loss. Additionally, we observed a reduced red-to-green fluorescence ratio in response to OHT in Mt-Keima reporter mice, indicating a disruption in autophagic flux prior to the onset of RGC degeneration. Elevated p62 levels, along with reduced phospho-ubiquitin (Ser65), suggest impaired recognition and clearance of damaged mitochondrial cargo under OHT conditions. Importantly, the unaltered expression of LAMP1 indicates that lysosomal integrity is preserved, pointing to a selective disruption in mitophagic flux rather than a general defect in lysosomal function. Notably, although these alterations were most prominent in RGCs, we also observed a modest increase in TOM20 levels in other retinal layers (non-significant) and decrease in p62 and LC3 levels within the outer plexiform layer (OPL) of GC-induced mouse model of glaucoma. These findings suggest that while the primary mitochondrial and autophagy defects occur in RGCs in a cell-autonomous manner, secondary changes in other retinal layers may reflect non-cell-autonomous responses associated with retinal stress during OHT.

The accumulation of damaged mitochondria and persistent oxidative stress due to defective mitophagy likely activate the intrinsic (mitochondrial) apoptotic pathway. This may promote the release of pro-apoptotic factors, such as cytochrome c, into the cytosol, which activate caspase-9 and caspase-3, ultimately leading to apoptosis of the RGC soma. Moreover, impaired energy production, calcium dysregulation, and neuroinflammation can further contribute to the structural and functional loss of RGCs, triggering neuronal degeneration. Glaucomatous neurodegeneration is compartmentalized, with distinct molecular signals contributing to the degeneration of both axons and somas [147]. Our study identifies impaired autophagy and mitophagy as a common pathological event underlying axonal degeneration and RGC soma loss.

Our findings further demonstrate that constitutive autophagy is essential for maintaining mitochondrial integrity, neuronal function, and the survival of RGCs. This is evidenced by the conditional knockdown of RGC-specific *Atg5*, which leads to neurodegeneration and is associated with an increased accumulation of damaged mitochondria and oxidative DNA damage. Interestingly, a previous study has reported that *Atg4b* knockout confers protection against OHT and neurodegeneration in both experimental and spontaneous glaucoma models [65]. These contrasting outcomes likely reflect fundamental differences in the roles of the autophagy-related genes involved. ATG5 is a core, non-redundant component of the autophagy machinery required for LC3 lipidation and autophagosome formation [148]. Consequently, its loss results in a complete disruption of autophagy/mitophagy, resulting in the accumulation of dysfunctional mitochondria and RGC death in our studies. In contrast, ATG4B belongs to a family of cysteine proteases with redundant isoforms that can partially compensate for its loss [63], thereby preserving autophagic flux and RGC viability.

Since autophagy is the fundamental mechanism for maintaining mitochondrial homeostasis, therapeutic interventions that induce autophagy may help ameliorate mitochondrial dysfunction and protect RGCs from IOP-induced damage. This study demonstrated that enhancing autophagy to clear the accumulated dysfunctional mitochondria is an attractive therapeutic strategy for glaucoma. We utilized Torin 2 to restore impaired autophagy in mouse models of glaucoma. Previously, we demonstrated that Torin 2 reversed TM cell dysfunction in a mouse model of myocilin POAG by enhancing the autophagic degradation of mutant myocilin. Torin 2-induced autophagy flux was associated with reduced mutant myocilin and p62 accumulation [66]. Consistent with this previously published study [66], Torin 2 enhanced mitophagy flux within 24 h of its administration (intravitreal) in Mt-Keima mitophagy reporter mice and reduced p62 accumulation in RGCL of OHT mice. Torin 2 is a second-generation mTOR catalytic inhibitor with higher potency than rapamycin and induces mitophagy more robustly by inhibiting both mTORC1 and 2 [149]. Torin 2 has been shown to improve tolerability significantly and exhibits therapeutic potential in the context of aging and neurodegenerative diseases [150]. One of the major strengths of our study is the demonstration of the therapeutic potential of autophagy enhancement via Torin 2 in mild to moderate neurodegenerative conditions. Importantly, enhancing mitophagy with Torin 2 after the onset of chronic OHT not only prevented neurodegeneration in mouse models of glaucoma but also promoted RGC survival in ex vivo human retinal explants, underscoring its potential as a disease-modifying therapy targeting mitochondrial turnover. Given the broad mTOR inhibition by Torin 2, our future studies will focus on more selective autophagy/mitophagy modulators or pathway-specific interventions to validate and refine mitophagy-targeted therapies for glaucoma. A limitation of our study, particularly in *Tg.Cre-MYOC*^*Y437H*^ mice, is that the RGC preservation observed with Torin 2 treatment may be partially attributable to IOP reduction. As demonstrated in this study and in our previous report [66], Torin2 promotes autophagic degradation of mutant myocilin, leading to reduced IOP. However, results from the GC-induced mouse model and ex vivo retinal explant experiments strongly support the notion that activation of autophagy itself can prevent glaucomatous neurodegeneration.

In conclusion, our study highlights that impaired autophagy plays a critical role in glaucomatous neurodegeneration, leading to the accumulation of damaged mitochondria and oxidative DNA damage, which contributes to RGC degeneration. autophagy impairment precedes neurodegeneration, indicating that it may serve as an early pathogenic event in the disease progression. Furthermore, the loss of autophagy resulted in the pathological accumulation of damaged mitochondria, ultimately leading to RGC death. In contrast, pharmacological activation of autophagy via Torin 2 mitigated glaucomatous damage, underscoring autophagy as a viable and promising therapeutic target for neuroprotection in glaucoma.

## Materials and methods

### Experimental animals

C57BL/6J were purchased from the Jackson Laboratory. *Atg*5^*flox/flox*^ mice on a pure C57BL/6J genetic background were provided by Dr. Noboru Mizushima (The University of Tokyo, Tokyo, Japan) and were received from Dr. Thomas Ferguson’s lab (Washington University School of Medicine, St. Louis, Missouri, USA). Mt-Keima mice on FVB/N genetic background were kindly provided by Dr. Toren Finkel (University of Pittsburgh). *Tg.Cre-MYOC*^*Y437H*^ mice on a pure C57BL/6J genetic background were developed in our laboratory as described recently [82]. Both male and female mice between the ages of 3 and 4 months were utilized in this study. Under the controlled conditions of temperature (21 to 26 °C) and humidity (40 to 70%), mice were subjected to a 12 h light/12 h dark cycle (8:00 PM to 8:00 AM). All animals were fed standard chow ad libitum and provided with water. The number of animals used in each experiment is indicated in the corresponding figure or figure legend.

### Mouse models of glaucoma

3-month-old mice were injected with 20 µL/eye of either Vehicle (Veh) or freshly prepared dexamethasone (Dex) (i.e. 200 µg/eye) suspension via the periocular route once a week for 5 or 10 weeks under isoflurane anesthetic conditions (isoflurane (2.5%); oxygen (0.8 L/min)). IOPs were monitored weekly. To induce *Tg.Cre-MYOC*^*Y437H*^ mice, 3-month-old-*Tg.Cre-MYOC*^*Y437H*^ mice were intravitreally injected with HAd5 expressing empty cassette or Cre (2 × 10^7^ pfu/eye) using a Hamilton syringe fitted with a sterile 33-gauge needle under isoflurane anesthetic conditions. IOPs were monitored weekly. At the end of the study period, mice were euthanized by inhalation of CO_2_ or intracardiac perfusion followed by cervical dislocation.

#### IOP measurements

IOPs were measured between 9 and 11 AM using a Tonolab rebound tonometry (Colonial Medical Supply) under isoflurane anesthesia as described previously [151, 152]. IOP measurements were performed once a week throughout the study period in a masked manner. At each time point, an average of six IOP readings were taken from each eye and represented as the final IOP value.

### Pattern electroretinography (PERG)

PERG measurements were performed using the Miami PERG system (Jorvec, Miami, FL) according to the manufacturer’s instructions to analyze the RGC function as described previously [80, 153]. Briefly, experimental and control mice were anesthetized with ketamine/xylazine solution (100/10 mg/kg) and placed on a temperature-controlled metal base at a fixed distance (10 cm) from the LED monitors to maintain a constant body temperature (37 °C). The PERG was derived simultaneously from each eye using electrodes placed subcutaneously at the snout (active), back of the head (reference), and tail (ground). An average of two consecutive repetitions was taken, and amplitudes (P1-N2) representing the RGC function were shown in the results.

### Immunostaining

Enucleated eyes from experimental and control mice were fixed in 4% paraformaldehyde for 3 h at room temperature and then processed and embedded in paraffin. Five-micron tissue sections were cut using a microtome, and immunostaining was performed as described previously [154, 155]. Briefly, tissue sections were deparaffinized in xylene and rehydrated in gradient concentrations of ethanol (100, 95, 70, and 50%), followed by antigen retrieval using citrate buffer (pH = 6). Following a 15-minute wash in 1X PBS, tissue sections were incubated with blocking buffer (containing 10% goat serum and 0.5%-2% Triton X-100 in 1X PBS) for 2 h, followed by overnight incubation with primary antibodies in blocking buffer at 4 °C. Following three washes in 1X PBS, tissue sections were further incubated with the appropriate secondary antibodies for 2 h at room temperature. Tissue sections were then washed again with 1X PBS and mounted with a mounting medium containing DAPI nuclear stain (Vector Labs, Inc., Burlingame, CA, USA). Images were captured by either a Keyence fluorescence microscope (Itasca, IL, USA) or a confocal microscope and analyzed using ImageJ. Tissue sections incubated without a primary antibody served as a negative control, and the relative signal intensities were subtracted from an averaged background intensity.

### Whole-mount retinal staining and RGC counting

The total number of surviving RGCs in experimental and control mice groups was determined by whole-mount staining with RGC-specific RBPMS antibody, as described previously [80, 153]. Briefly, the eyes were enucleated and fixed in 4% PFA for 12 h at 4 °C. The eyes were then rinsed with 1X PBS. The anterior segment was removed, and the whole retina was carefully separated from the posterior cup. The isolated retinas were incubated at 4 °C for 12 h in blocking buffer (10% goat serum and 0.2% Triton X-100 in PBS). The whole retinas were then incubated with RBPMS antibody for three days at 4 °C and washed 3 times in 1x PBS. The retinas were further incubated with a corresponding secondary antibody (goat anti-rabbit 568, 1:500; Invitrogen) for 2 h at room temperature, rinsed 3 times in 1X PBS, and flat-mounted on a glass slide by cutting four equal quadrants attached to the center of the retina. A Keyence microscope was used to capture the images at a magnification of 20x. For counting RGCs, non-overlapping images from the entire retina, including the periphery, mid-periphery, and center of the retina, were taken, and RBPMS-positive cells were counted using ImageJ software.

### Optic nerve axonal damage by paraphenylenediamine (PPD) staining and TEM analysis

ON axonal degeneration from experimental and control mice groups was examined using PPD staining. Transmission electron microscopy (TEM) was used to further examine the ultrastructural morphology of degenerative axons. ON were incubated overnight with 1% osmium tetroxide at 4 °C and then rinsed with 0.1 M phosphate buffer and 0.1 M sodium-acetate buffer, followed by dehydration in graded ethanol concentrations. ON was embedded in resin (Eponate-12; Ted Pella), and semithin sections were cut and stained with 1% PPD for 10 min after embedding. On each ON, five images were taken without overlap with a Zeiss confocal microscope, and surviving axons were counted in an area of 8100 square micrometers from each image using Image J software. TEM analysis was performed on 1 μm sections of ON from experimental and control mice groups at the Electron Microscopy Core Facility at the University of Missouri, Columbia.

### Analysis of ex vivo mitophagy flux

ON from Mt-Keima mice were carefully removed and immediately sealed under the coverslip on a glass slide and analyzed using confocal microscopy with excitations at 561 (red) and 458 nm (green). The level of mitophagy flux was calculated by dividing the area of the “red” signal (excitation by wavelength 561 nm) by the area of the “green” signal (excitation by wavelength 458 nm), using the Fiji software package, which is based on ImageJ (National Institutes of Health).

#### Ex vivo **culture of human axotomized retinal explants**

Human retinas were isolated from healthy donors within 12 h of their death from the UNTHSC Willed Body Program. Inclusion criteria include healthy donor eyes that are free from any ocular diseases and other neurodegenerative conditions. A total of six to eight explants or four equal retinal quadrants were isolated from each healthy retina and placed on Transwell Permeable 6.5 mm inserts or in 12 well plates with the RGC layer facing up. The retinal explants and quadrants were cultured for 7 days under neurotrophic factors deprivation conditions and treated either with DMSO or Torin 2 (100 µM) in an explant medium consisting of phenol red-free Neurobasal A with 2% B-27, 1% N-2, 0.8 mM L-glutamine, 100 U/mL penicillin, and 100 µg/mL streptomycin (all reagents were purchased from Thermo Fisher Scientific, Waltham, MA, USA). Following the 7-day treatment, the retinal explants were fixed with 4% PFA for 24 h at 4 °C and then washed 3 times in 1X PBS. All explants were blocked with blocking buffer (10% goat serum, 0.2% Triton X-100 in 1X PBS) for at least 2 h at room temperature and then incubated with RBPMS antibody for three days at 4 °C. Following washing 3 washes in 1X PBS, the retinal explants were further incubated with a corresponding secondary antibody (goat anti-rabbit 568, 1:500; Invitrogen) for 2 h at room temperature. The retinal explants were rinsed 3 times in 1X PBS and mounted with DAPI mounting solution on glass slides. Images were captured using a Keyence microscope, and RBPMS-positive cells were counted using ImageJ software. For biochemical analysis, retinal explant lysates were collected after 24 h of treatment and subjected to Western blot analysis for p62, with GAPDH used as a loading control.

### Treatment of mice with Torin 2

Three-month-old Mt-Keima mice were intravitreally injected with Torin 2 (1 mM, prepared in DMSO) in one eye, while the contralateral eye received DMSO as control under isoflurane anesthesia. Mice were euthanized 24 h after treatment, and mitophagy flux was measured ex vivo in the ON.

In C57BL/6J mice, IOP was elevated for 7 weeks using weekly periocular injections of Dex. Mice then received intravitreal Torin 2 (1 mM, prepared in DMSO) in one eye and DMSO in the contralateral eye. After 3 weeks under sustained IOP elevation, mice were euthanized and the structural and functional integrity of RGCs and their axons was assessed. In a subset of Dex-induced OHT mice treated with Torin 2, cholera toxin B (CTB) was intravitreally injected to assess axonal transport. Forty-eight hours after CTB injection, anterograde axonal transport to the superior colliculus was evaluated by dorsal imaging of the mouse brain.

To further evaluate the effect of Torin 2, 3-month-old *Tg.Cre-MYOC*^*Y437H*^ mice were induced by intravitreal injection of HAd5 expressing either empty cassette or Cre (2 × 10⁷ pfu/eye). Following 7 weeks of IOP elevation, mice received intravitreal Torin 2 (1 mM in DMSO) in one eye and DMSO in the contralateral eye. Three weeks later, mice were euthanized and RGC structural and functional integrity were assessed.

### Adeno-Associated Viral 2 (AAV2) vector injections

AAV2 vectors expressing empty/null or Cre under the control of a synapsin (Syn) promoter were purchased from SignaGen Laboratories. For RGC-specific conditional knockdown of *Atg*5, three-month-old *Atg5*^*flox/flox*^ mice were injected intravitreally one eye with AAV2-Syn-Cre, and the contralateral eyes were injected with AAV2-Syn-empty vector using a Hamilton syringe fitted with a sterile 33-gauge needle. Following 6 weeks post-injection (a time point chosen to allow efficient AAV-mediated gene expression in RGCs), the mice were euthanized, and RGC-specific *Atg*5 knockdown and glaucoma phenotypes were examined by immunostaining of retinal cross-sections.

### Antibodies and reagents

Antibodies and reagents were purchased from the following sources: RBPMS (catalog # 118619, Gene Tex and catalog #PA5-11 9676, Invitrogen), TOMM20 (catalog # sc-17764 from Santa Cruz and catalog # 11802 from Proteintech Inc), CoxIV (catalog # 11242-1, Proteintech), SOD2 (catalog # ab16956, Abcam), 8-OHdG (catalog # sc-66036, Santa Cruz), p62 (catalog # PM045, MBL), LC3 (catalog # GTX127375, Gene Tex), LC3B (E5Q2K) (catalog #83506, CST), LAMP1 (D4O1S) (catalog #15665, CST), Phospho-Ubiquitin (Ser65) (E5T1W) (catalog #70973, CST), Annexin V (catalog # NBP2-52414, Novus), GAPDH (catalog # PA1-987-HRP, Invitrogen), TUJ1 (catalog # GTX130245, Genetex), Cholera Toxin B (catalog # C34776, Invitrogen). Goat anti-Rabbit IgG (H + L) Cross-Adsorbed Secondary Antibody, Alexa Fluor™ 568 (catalog # A-11011, Invitrogen), Goat anti-Rabbit IgG (H + L) Highly Cross-Adsorbed Secondary Antibody, Alexa Fluor™ 488 (catalog # A-11034, Invitrogen), Goat anti-Rabbit IgG (H + L) Cross-Adsorbed Secondary Antibody, Alexa Fluor™ 680 (catalog #A-21076, Invitrogen), Goat anti-Mouse IgG (H + L) Highly Cross-Adsorbed Secondary Antibody, Alexa Fluor™ 568 (catalog # A-11031, Invitrogen), Goat anti-Mouse IgG (H + L) Cross-Adsorbed Secondary Antibody, Alexa Fluor™ 488 (catalog # A-11001, Invitrogen). Dexamethasone, Micronized, USP (Spectrum, DE121), Torin 2 (Millipore Sigma, SML 1224), 16% PFA Aqueous Solution EM Grade (Electron Microscopy Sciences, 15710-S), Aqueous Glutaraldehyde EM Grade, 10% (Electron Microscopy Sciences, SKU 16120), Sodium Cacodylate Buffer, Electron Microscopy Sciences (catalog # 102090-962, Avantor), DAPI (VECTASHIELD Antifade Mounting Medium, Vector Laboratories).

### Statistical analysis

GraphPad Prism v10.0 software was used for statistical analysis. N refers to the total number of eyes used for the study. Data was represented as mean ± SD. For comparing two different study groups, we used a 2-tailed Student’s t-test with or without Welch’s correction. For comparing three study groups, a 2-way ANOVA with multiple comparisons was employed. A value of *p* < 0.05 was considered statistically significant.

### Scientific illustration

BioRender was used to create the illustrations for this article.

### Sex as a biological variable

Animals of both sexes were included in the study; however, sex-specific differences were not explicitly analyzed or incorporated into the data interpretation. 

## Supplementary Information

Below is the link to the electronic supplementary material.

Supplementary Material 1

Supplementary Material 2

Supplementary Material 3

## Data Availability

All data supporting the findings of this study are available within the paper and its Supplementary Information.

## Declarations

### Study approval

Animal studies were conducted in compliance with the Association for Research in Vision and Ophthalmology Statement on the Use of Animals in Ophthalmic and Vision Research and the Institutional Animal Care and Use Committee regulations of the University of North Texas Health Science Center (approved protocol: IACUC2018-0032 and IACUC2020-0026), University of Missouri (approved protocol: ACUC 44632) and University of California Irvine (approved protocol: AUP-22-159).

## References

[1] Tham YC, Li X, Wong TY, Quigley HA, Aung T, Cheng CY. Global prevalence of glaucoma and projections of glaucoma burden through 2040: a systematic review and meta-analysis. Ophthalmology. 2014;121(11):2081–90. 24974815 10.1016/j.ophtha.2014.05.013

[2] Allison K, Patel D, Alabi O. Epidemiology of Glaucoma: The Past, Present, and Predictions for the Future. Cureus. 2020;12(11):e11686. 33391921 10.7759/cureus.11686 PMC7769798

[3] Zhao L, Li J, Feng L, Zhang C, Zhang W, Wang C, et al. Depicting Developing Trend and Core Knowledge of Primary Open-Angle Glaucoma: A Bibliometric and Visualized Analysis. Front Med (Lausanne). 2022;9:922527. 35865166 10.3389/fmed.2022.922527 PMC9294470

[4] Gabelt BT, Kaufman PL. Changes in aqueous humor dynamics with age and glaucoma. Prog Retin Eye Res. 2005;24(5):612–37. 15919228 10.1016/j.preteyeres.2004.10.003

[5] Li G, van Batenburg-Sherwood J, Safa BN, Fraticelli Guzman NS, Wilson A, Bahrani Fard MR, et al. Aging and intraocular pressure homeostasis in mice. Aging Cell. 2024;23(7):e14160. 38566432 10.1111/acel.14160 PMC11258442

[6] Burgoyne CF. A biomechanical paradigm for axonal insult within the optic nerve head in aging and glaucoma. Exp Eye Res. 2011;93(2):120–32. 20849846 10.1016/j.exer.2010.09.005 PMC3128181

[7] Howell GR, Libby RT, Jakobs TC, Smith RS, Phalan FC, Barter JW, et al. Axons of retinal ganglion cells are insulted in the optic nerve early in DBA/2J glaucoma. J Cell Biol. 2007;179(7):1523–37. 18158332 10.1083/jcb.200706181 PMC2373494

[8] Crish SD, Sappington RM, Inman DM, Horner PJ, Calkins DJ. Distal axonopathy with structural persistence in glaucomatous neurodegeneration. Proc Natl Acad Sci U S A. 2010;107(11):5196–201. 20194762 10.1073/pnas.0913141107 PMC2841892

[9] Fernandez-Albarral JA, Ramirez AI, de Hoz R, Matamoros JA, Salobrar-Garcia E, Elvira-Hurtado L, et al. Glaucoma: from pathogenic mechanisms to retinal glial cell response to damage. Front Cell Neurosci. 2024;18:1354569. 38333055 10.3389/fncel.2024.1354569 PMC10850296

[10] Williams PA, Marsh-Armstrong N, Howell GR. Lasker IIoA, Glaucomatous Neurodegeneration P. Neuroinflammation in glaucoma: A new opportunity. Exp Eye Res. 2017;157:20–7. 28242160 10.1016/j.exer.2017.02.014 PMC5497582

[11] Calkins DJ. Adaptive responses to neurodegenerative stress in glaucoma. Prog Retin Eye Res. 2021;84:100953. 33640464 10.1016/j.preteyeres.2021.100953 PMC8384979

[12] Inman DM, Harun-Or-Rashid M. Metabolic Vulnerability in the Neurodegenerative Disease Glaucoma. Front Neurosci. 2017;11:146. 28424571 10.3389/fnins.2017.00146 PMC5371671

[13] Yang X, Li S, Guo H, Wang S, Sun H, Wang J, et al. Metabolic dysregulation in glaucoma. Clin Exp Optom. 2025;1–7. 10.1080/08164622.2025.2463502 39938920

[14] Rao M, Huang YK, Liu CC, Meadows C, Cheng HC, Zhou M, et al. Aldose reductase inhibition decelerates optic nerve degeneration by alleviating retinal microglia activation. Sci Rep. 2023;13(1):5592. 37019993 10.1038/s41598-023-32702-5 PMC10076364

[15] Canadian Glaucoma Study G. Canadian Glaucoma Study: 1. Study design, baseline characteristics, and preliminary analyses. Can J Ophthalmol. 2006;41(5):566–75. 17016527 10.1139/i06-057

[16] Heijl A, Buchholz P, Norrgren G, Bengtsson B. Rates of visual field progression in clinical glaucoma care. Acta Ophthalmol. 2013;91(5):406–12. 23066646 10.1111/j.1755-3768.2012.02492.x PMC3798127

[17] Heijl A, Leske MC, Bengtsson B, Hyman L, Bengtsson B, Hussein M, et al. Reduction of intraocular pressure and glaucoma progression: results from the Early Manifest Glaucoma Trial. Arch Ophthalmol. 2002;120(10):1268–79. 12365904 10.1001/archopht.120.10.1268

[18] Sheybani A, Scott R, Samuelson TW, Kahook MY, Bettis DI, Ahmed IIK, et al. Open-Angle Glaucoma: Burden of Illness, Current Therapies, and the Management of Nocturnal IOP Variation. Ophthalmol Ther. 2020;9(1):1–14. 31732872 10.1007/s40123-019-00222-z PMC7054505

[19] Calkins DJ. Critical pathogenic events underlying progression of neurodegeneration in glaucoma. Prog Retin Eye Res. 2012;31(6):702–19. 22871543 10.1016/j.preteyeres.2012.07.001 PMC3472111

[20] Nickells RW. Retinal ganglion cell death in glaucoma: the how, the why, and the maybe. J Glaucoma. 1996;5(5):345–56. 8897235

[21] Quigley HA, Dunkelberger GR, Green WR. Retinal ganglion cell atrophy correlated with automated perimetry in human eyes with glaucoma. Am J Ophthalmol. 1989;107(5):453–64. 2712129 10.1016/0002-9394(89)90488-1

[22] Ito YA, Di Polo A. Mitochondrial dynamics, transport, and quality control: A bottleneck for retinal ganglion cell viability in optic neuropathies. Mitochondrion. 2017;36:186–92. 28866056 10.1016/j.mito.2017.08.014

[23] Balog J, Mehta SL, Vemuganti R. Mitochondrial fission and fusion in secondary brain damage after CNS insults. J Cereb Blood Flow Metab. 2016;36(12):2022–33. 27677674 10.1177/0271678X16671528 PMC5363672

[24] Yokota S, Shah SH, Huie EL, Wen RR, Luo Z, Goldberg JL. Kif5a Regulates Mitochondrial Transport in Developing Retinal Ganglion Cells In Vitro. Invest Ophthalmol Vis Sci. 2023;64(3):4. 36862119 10.1167/iovs.64.3.4 PMC9983700

[25] Kreymerman A, Buickians DN, Nahmou MM, Tran T, Galvao J, Wang Y, et al. MTP18 is a Novel Regulator of Mitochondrial Fission in CNS Neuron Development, Axonal Growth, and Injury Responses. Sci Rep. 2019;9(1):10669. 31337818 10.1038/s41598-019-46956-5 PMC6650498

[26] Shah SH, Goldberg JL. The Role of Axon Transport in Neuroprotection and Regeneration. Dev Neurobiol. 2018;78(10):998–1010. 30027690 10.1002/dneu.22630 PMC8326243

[27] Osborne NN, Nunez-Alvarez C, Joglar B, Del Olmo-Aguado S, Glaucoma. Focus on mitochondria in relation to pathogenesis and neuroprotection. Eur J Pharmacol. 2016;787:127–33. 27090928 10.1016/j.ejphar.2016.04.032

[28] Eells JT. Mitochondrial dysfunction in the aging retina. Biology (Basel). 2019;8(2). 10.3390/biology8020031 PMC6627398 31083549

[29] Muench NA, Patel S, Maes ME, Donahue RJ, Ikeda A, Nickells RW. The influence of mitochondrial dynamics and function on retinal ganglion cell susceptibility in optic nerve disease. Cells. 2021;10(7). 10.3390/cells10071593 PMC8306483 34201955

[30] Osborne NN, Nunez-Alvarez C, Del Olmo-Aguado S. The effect of visual blue light on mitochondrial function associated with retinal ganglions cells. Exp Eye Res. 2014;128:8–14. 25193034 10.1016/j.exer.2014.08.012

[31] Moreira PI, Zhu X, Wang X, Lee HG, Nunomura A, Petersen RB, et al. Mitochondria: a therapeutic target in neurodegeneration. Biochim Biophys Acta. 2010;1802(1):212–20. 19853657 10.1016/j.bbadis.2009.10.007 PMC2790540

[32] Rangaraju V, Lewis TL Jr., Hirabayashi Y, Bergami M, Motori E, Cartoni R, et al. Pleiotropic Mitochondria: The Influence of Mitochondria on Neuronal Development and Disease. J Neurosci. 2019;39(42):8200–8. 31619488 10.1523/JNEUROSCI.1157-19.2019 PMC6794931

[33] Lou G, Palikaras K, Lautrup S, Scheibye-Knudsen M, Tavernarakis N, Fang EF. Mitophagy and Neuroprotection. Trends Mol Med. 2020;26(1):8–20. 31375365 10.1016/j.molmed.2019.07.002

[34] Khalil B, El Fissi N, Aouane A, Cabirol-Pol MJ, Rival T, Lievens JC. PINK1-induced mitophagy promotes neuroprotection in Huntington’s disease. Cell Death Dis. 2015;6(1):e1617. 25611391 10.1038/cddis.2014.581 PMC4669776

[35] Hass DT, Barnstable CJ. Mitochondrial Uncoupling Protein 2 Knock-out Promotes Mitophagy to Decrease Retinal Ganglion Cell Death in a Mouse Model of Glaucoma. J Neurosci. 2019;39(18):3582–96. 30814312 10.1523/JNEUROSCI.2702-18.2019 PMC6495138

[36] Coughlin L, Morrison RS, Horner PJ, Inman DM. Mitochondrial morphology differences and mitophagy deficit in murine glaucomatous optic nerve. Invest Ophthalmol Vis Sci. 2015;56(3):1437–46. 25655803 10.1167/iovs.14-16126 PMC4347310

[37] Chrysostomou V, Rezania F, Trounce IA, Crowston JG. Oxidative stress and mitochondrial dysfunction in glaucoma. Curr Opin Pharmacol. 2013;13(1):12–5. 23069478 10.1016/j.coph.2012.09.008

[38] Kamel K, Farrell M, O’Brien C. Mitochondrial dysfunction in ocular disease: Focus on glaucoma. Mitochondrion. 2017;35:44–53. 28499981 10.1016/j.mito.2017.05.004

[39] Kong GY, Van Bergen NJ, Trounce IA, Crowston JG. Mitochondrial dysfunction and glaucoma. J Glaucoma. 2009;18(2):93–100. 19225343 10.1097/IJG.0b013e318181284f

[40] Lascaratos G, Garway-Heath DF, Willoughby CE, Chau KY, Schapira AH. Mitochondrial dysfunction in glaucoma: understanding genetic influences. Mitochondrion. 2012;12(2):202–12. 22138560 10.1016/j.mito.2011.11.004

[41] Leruez S, Marill A, Bresson T, de Saint Martin G, Buisset A, Muller J, et al. A Metabolomics Profiling of Glaucoma Points to Mitochondrial Dysfunction, Senescence, and Polyamines Deficiency. Invest Ophthalmol Vis Sci. 2018;59(11):4355–61. 30193307 10.1167/iovs.18-24938

[42] Osborne NN, Alvarez CN, del Olmo Aguado S. Targeting mitochondrial dysfunction as in aging and glaucoma. Drug Discov Today. 2014;19(10):1613–22. 24880106 10.1016/j.drudis.2014.05.010

[43] Yu-Wai-Man P. Mitochondrial dysfunction in glaucoma: closing the loop. Invest Ophthalmol Vis Sci. 2012;53(4):2438. 22547661 10.1167/iovs.12-9815

[44] Abu-Amero KK, Morales J, Bosley TM. Mitochondrial abnormalities in patients with primary open-angle glaucoma. Invest Ophthalmol Vis Sci. 2006;47(6):2533–41. 16723467 10.1167/iovs.05-1639

[45] Sundaresan P, Simpson DA, Sambare C, Duffy S, Lechner J, Dastane A, et al. Whole-mitochondrial genome sequencing in primary open-angle glaucoma using massively parallel sequencing identifies novel and known pathogenic variants. Genet Med. 2015;17(4):279–84. 25232845 10.1038/gim.2014.121

[46] Mohanty K, Mishra S, Dada R, Dada T. Mitochondrial Genome Alterations, Cytochrome C Oxidase Activity, and Oxidative Stress: Implications in Primary Open-angle Glaucoma. J Curr Glaucoma Pract. 2022;16(3):158–65. 36793267 10.5005/jp-journals-10078-1376 PMC9905874

[47] Collins DW, Gudiseva HV, Trachtman B, Bowman AS, Sagaser A, Sankar P, et al. Association of primary open-angle glaucoma with mitochondrial variants and haplogroups common in African Americans. Mol Vis. 2016;22:454–71. 27217714 PMC4872278

[48] Ju WK, Liu Q, Kim KY, Crowston JG, Lindsey JD, Agarwal N, et al. Elevated hydrostatic pressure triggers mitochondrial fission and decreases cellular ATP in differentiated RGC-5 cells. Invest Ophthalmol Vis Sci. 2007;48(5):2145–51. 17460273 10.1167/iovs.06-0573

[49] Cheng XT, Huang N, Sheng ZH. Programming axonal mitochondrial maintenance and bioenergetics in neurodegeneration and regeneration. Neuron. 2022;110(12):1899–923. 35429433 10.1016/j.neuron.2022.03.015 PMC9233091

[50] Han S, Zhang M, Jeong YY, Margolis DJ, Cai Q. The role of mitophagy in the regulation of mitochondrial energetic status in neurons. Autophagy. 2021;17(12):4182–201. 33757395 10.1080/15548627.2021.1907167 PMC8726713

[51] Yang TH, Kang EY, Lin PH, Yu BB, Wang JH, Chen V, et al. Mitochondria in retinal ganglion cells: unraveling the metabolic nexus and oxidative stress. Int J Mol Sci. 2024;25(16). 10.3390/ijms25168626 PMC11354650 39201313

[52] Carelli V, La Morgia C, Ross-Cisneros FN, Sadun AA. Optic neuropathies: the tip of the neurodegeneration iceberg. Hum Mol Genet. 2017;26(R2):R139–50. 28977448 10.1093/hmg/ddx273 PMC5886475

[53] Williams PA, Harder JM, Foxworth NE, Cochran KE, Philip VM, Porciatti V, et al. Vitamin B(3) modulates mitochondrial vulnerability and prevents glaucoma in aged mice. Science. 2017;355(6326):756–60. 28209901 10.1126/science.aal0092 PMC5408298

[54] Rodriguez-Muela N, Germain F, Marino G, Fitze PS, Boya P. Autophagy promotes survival of retinal ganglion cells after optic nerve axotomy in mice. Cell Death Differ. 2012;19(1):162–9. 21701497 10.1038/cdd.2011.88 PMC3252838

[55] Ma K, Chen G, Li W, Kepp O, Zhu Y, Chen Q. Mitophagy, Mitochondrial Homeostasis, and Cell Fate. Front Cell Dev Biol. 2020;8:467. 32671064 10.3389/fcell.2020.00467 PMC7326955

[56] Kissova I, Salin B, Schaeffer J, Bhatia S, Manon S, Camougrand N. Selective and non-selective autophagic degradation of mitochondria in yeast. Autophagy. 2007;3(4):329–36. 17377488 10.4161/auto.4034

[57] Rasbach KA, Schnellmann RG. Signaling of mitochondrial biogenesis following oxidant injury. J Biol Chem. 2007;282(4):2355–62. 17116659 10.1074/jbc.M608009200

[58] Mizushima N, Komatsu M. Autophagy: renovation of cells and tissues. Cell. 2011;147(4):728–41. 22078875 10.1016/j.cell.2011.10.026

[59] Pankiv S, Clausen TH, Lamark T, Brech A, Bruun JA, Outzen H, et al. p62/SQSTM1 binds directly to Atg8/LC3 to facilitate degradation of ubiquitinated protein aggregates by autophagy. J Biol Chem. 2007;282(33):24131–45. 17580304 10.1074/jbc.M702824200

[60] Narendra D, Tanaka A, Suen DF, Youle RJ. Parkin is recruited selectively to impaired mitochondria and promotes their autophagy. J Cell Biol. 2008;183(5):795–803. 19029340 10.1083/jcb.200809125 PMC2592826

[61] Green DR, Galluzzi L, Kroemer G. Mitochondria and the autophagy-inflammation-cell death axis in organismal aging. Science. 2011;333(6046):1109–12. 21868666 10.1126/science.1201940 PMC3405151

[62] Pickrell AM, Youle RJ. The roles of PINK1, parkin, and mitochondrial fidelity in Parkinson’s disease. Neuron. 2015;85(2):257–73. 25611507 10.1016/j.neuron.2014.12.007 PMC4764997

[63] Agrotis A, Pengo N, Burden JJ, Ketteler R. Redundancy of human ATG4 protease isoforms in autophagy and LC3/GABARAP processing revealed in cells. Autophagy. 2019;15(6):976–97. 30661429 10.1080/15548627.2019.1569925 PMC6526816

[64] Bell K, Rosignol I, Sierra-Filardi E, Rodriguez-Muela N, Schmelter C, Cecconi F, et al. Age related retinal Ganglion cell susceptibility in context of autophagy deficiency. Cell Death Discov. 2020;6:21. 32337073 10.1038/s41420-020-0257-4 PMC7165178

[65] Dixon A, Shim MS, Nettesheim A, Coyne A, Su CC, Gong H, et al. Autophagy deficiency protects against ocular hypertension and neurodegeneration in experimental and spontanous glaucoma mouse models. Cell Death Dis. 2023;14(8):554. 37620383 10.1038/s41419-023-06086-3 PMC10449899

[66] Kasetti RB, Maddineni P, Kiehlbauch C, Patil S, Searby CC, Levine B, et al. Autophagy stimulation reduces ocular hypertension in a murine glaucoma model via autophagic degradation of mutant myocilin. JCI Insight. 2021;6(5). 10.1172/jci.insight.143359 PMC8021112 33539326

[67] Nettesheim A, Dixon A, Shim MS, Coyne A, Walsh M, Liton PB. Autophagy in the Aging and Experimental Ocular Hypertensive Mouse Model. Invest Ophthalmol Vis Sci. 2020;61(10):31. 32797200 10.1167/iovs.61.10.31 PMC7441338

[68] Park HY, Kim JH, Park CK. Activation of autophagy induces retinal ganglion cell death in a chronic hypertensive glaucoma model. Cell Death Dis. 2012;3(4):e290. 22476098 10.1038/cddis.2012.26 PMC3358006

[69] Cai Q, Tammineni P. Alterations in Mitochondrial Quality Control in Alzheimer’s Disease. Front Cell Neurosci. 2016;10:24. 26903809 10.3389/fncel.2016.00024 PMC4746252

[70] Chakravorty A, Jetto CT, Manjithaya R. Dysfunctional Mitochondria and Mitophagy as Drivers of Alzheimer’s Disease Pathogenesis. Front Aging Neurosci. 2019;11:311. 31824296 10.3389/fnagi.2019.00311 PMC6880761

[71] D’Urso B, Weil R, Genin P. [Optineurin and mitochondrial dysfunction in neurodegeneration]. Med Sci (Paris). 2024;40(2):167–75. 38411425 10.1051/medsci/2023220

[72] Ju WK, Perkins GA, Kim KY, Bastola T, Choi WY, Choi SH. Glaucomatous optic neuropathy: Mitochondrial dynamics, dysfunction and protection in retinal ganglion cells. Prog Retin Eye Res. 2023;95:101136. 36400670 10.1016/j.preteyeres.2022.101136

[73] Khacho M, Clark A, Svoboda DS, MacLaurin JG, Lagace DC, Park DS, et al. Mitochondrial dysfunction underlies cognitive defects as a result of neural stem cell depletion and impaired neurogenesis. Hum Mol Genet. 2017;26(17):3327–41. 28595361 10.1093/hmg/ddx217 PMC5886206

[74] Kuang G, Halimitabrizi M, Edziah AA, Salowe R, O’Brien JM. The potential for mitochondrial therapeutics in the treatment of primary open-angle glaucoma: a review. Front Physiol. 2023;14:1184060. 37601627 10.3389/fphys.2023.1184060 PMC10433652

[75] Lin MM, Liu N, Qin ZH, Wang Y. Mitochondrial-derived damage-associated molecular patterns amplify neuroinflammation in neurodegenerative diseases. Acta Pharmacol Sin. 2022;43(10):2439–47. 35233090 10.1038/s41401-022-00879-6 PMC9525705

[76] Luo H, Lai Y, Tang W, Wang G, Shen J, Liu H. Mitochondrial transplantation: a promising strategy for treating degenerative joint diseases. J Transl Med. 2024;22(1):941. 39407249 10.1186/s12967-024-05752-0 PMC11475785

[77] Misrani A, Tabassum S, Yang L. Mitochondrial Dysfunction and Oxidative Stress in Alzheimer’s Disease. Front Aging Neurosci. 2021;13:617588. 33679375 10.3389/fnagi.2021.617588 PMC7930231

[78] Venkatesan A, Ridilla M, Castro N, Wolosin JM, Henty-Ridilla JL, Knox BE, et al. Mitochondrial and microtubule defects in exfoliation glaucoma. bioRxiv. 2024. 10.1016/j.freeradbiomed.2025.03.046 PMC12074171 40180018

[79] Zhou DB, Castanos MV, Geyman L, Rich CA, Tantraworasin A, Ritch R, et al. Mitochondrial Dysfunction in Primary Open-Angle Glaucoma Characterized by Flavoprotein Fluorescence at the Optic Nerve Head. Ophthalmol Glaucoma. 2022;5(4):413–20. 34968754 10.1016/j.ogla.2021.12.006

[80] Maddineni P, Kasetti RB, Patel PD, Millar JC, Kiehlbauch C, Clark AF, et al. CNS axonal degeneration and transport deficits at the optic nerve head precede structural and functional loss of retinal ganglion cells in a mouse model of glaucoma. Mol Neurodegener. 2020;15(1):48. 32854767 10.1186/s13024-020-00400-9 PMC7457267

[81] Patel GC, Phan TN, Maddineni P, Kasetti RB, Millar JC, Clark AF, et al. Dexamethasone-Induced Ocular Hypertension in Mice: Effects of Myocilin and Route of Administration. Am J Pathol. 2017;187(4):713–23. 28167045 10.1016/j.ajpath.2016.12.003 PMC5397678

[82] Kaipa BR, Kasetti R, Li L, Millar JC, Cho W, Skowronska-Krawczyk D, et al. Ocular hypertension impairs axonal transport in the optic nerve head leading to neurodegeneration in a novel cre-inducible mouse model of myocilin glaucoma. bioRxiv. 2024.

[83] Kaipa BR, Kasetti R, Sundaresan Y, Li L, Yacoub S, Millar JC, et al. Impaired axonal transport contributes to neurodegeneration in a Cre-inducible mouse model of myocilin-associated glaucoma. JCI Insight. 2025. 10.1172/jci.insight.188710 PMC11949003 39836483

[84] Maddineni P, Kasetti RB, Kodati B, Yacoub S, Zode GS. Sodium 4-phenylbutyrate reduces ocular hypertension by degrading extracellular matrix deposition via activation of MMP9. Int J Mol Sci. 2021;22(18). 10.3390/ijms221810095 PMC8465971 34576258

[85] Maddineni P, Sundaresan Y, Zode G. Mouse Model of Glucocorticoid-Induced Glaucoma. Methods Mol Biol. 2025;2858:131–41. 39433673 10.1007/978-1-0716-4140-8_12

[86] Sun N, Yun J, Liu J, Malide D, Liu C, Rovira II, et al. Measuring Vivo Mitophagy Mol Cell. 2015;60(4):685–96. 26549682 10.1016/j.molcel.2015.10.009 PMC4656081

[87] Martinez-Vicente M. Neuronal Mitophagy in Neurodegenerative Diseases. Front Mol Neurosci. 2017;10:64. 28337125 10.3389/fnmol.2017.00064 PMC5340781

[88] D’Amico AG, Maugeri G, Magri B, Bucolo C, D’Agata V. Targeting the PINK1/Parkin pathway: A new perspective in the prevention and therapy of diabetic retinopathy. Exp Eye Res. 2024;247:110024. 39117133 10.1016/j.exer.2024.110024

[89] Huang Z, Ren S, Jiang Y, Wang T. PINK1 and Parkin cooperatively protect neurons against constitutively active TRP channel-induced retinal degeneration in Drosophila. Cell Death Dis. 2016;7(4):e2179. 27054334 10.1038/cddis.2016.82 PMC4855661

[90] Watzlawik JO, Hou X, Richardson T, Lewicki SL, Siuda J, Wszolek ZK, et al. Development and characterization of phospho-ubiquitin antibodies to monitor PINK1-PRKN signaling in cells and tissue. Autophagy. 2024;20(9):2076–91. 38802071 10.1080/15548627.2024.2356490 PMC11346534

[91] Harper JW, Ordureau A, Heo JM. Building and decoding ubiquitin chains for mitophagy. Nat Rev Mol Cell Biol. 2018;19(2):93–108. 29358684 10.1038/nrm.2017.129

[92] Kane LA, Lazarou M, Fogel AI, Li Y, Yamano K, Sarraf SA, et al. PINK1 phosphorylates ubiquitin to activate Parkin E3 ubiquitin ligase activity. J Cell Biol. 2014;205(2):143–53. 24751536 10.1083/jcb.201402104 PMC4003245

[93] Kazlauskaite A, Kondapalli C, Gourlay R, Campbell DG, Ritorto MS, Hofmann K, et al. Parkin is activated by PINK1-dependent phosphorylation of ubiquitin at Ser65. Biochem J. 2014;460(1):127–39. 24660806 10.1042/BJ20140334 PMC4000136

[94] Ordureau A, Sarraf SA, Duda DM, Heo JM, Jedrychowski MP, Sviderskiy VO, et al. Quantitative proteomics reveal a feedforward mechanism for mitochondrial PARKIN translocation and ubiquitin chain synthesis. Mol Cell. 2014;56(3):360–75. 25284222 10.1016/j.molcel.2014.09.007 PMC4254048

[95] Palmer JE, Wilson N, Son SM, Obrocki P, Wrobel L, Rob M, et al. Autophagy, aging, and age-related neurodegeneration. Neuron. 2024. 10.1016/j.neuron.2024.09.015 39406236

[96] Belousov DM, Mikhaylenko EV, Somasundaram SG, Kirkland CE, Aliev G. The Dawn of Mitophagy: What Do We Know by Now? Curr Neuropharmacol. 2021;19(2):170–92. 32442087 10.2174/1570159X18666200522202319 PMC8033973

[97] Galluzzi L, Baehrecke EH, Ballabio A, Boya P, Bravo-San Pedro JM, Cecconi F, et al. Molecular definitions of autophagy and related processes. EMBO J. 2017;36(13):1811–36. 28596378 10.15252/embj.201796697 PMC5494474

[98] Klionsky DJ, Abdel-Aziz AK, Abdelfatah S, Abdellatif M, Abdoli A, Abel S, et al. Guidelines for the use and interpretation of assays for monitoring autophagy (4th edition)(1). Autophagy. 2021;17(1):1-382. 10.1080/15548627.2020.1797280 PMC7996087 33634751

[99] Klionsky DJ, Abdelmohsen K, Abe A, Abedin MJ, Abeliovich H, Acevedo Arozena A, et al. Guidelines for the use and interpretation of assays for monitoring autophagy (3rd edition). Autophagy. 2016;12(1):1-222. 10.1080/15548627.2015.1100356 PMC4835977 26799652

[100] Kaipa BR, Kasetti R, Sundaresan Y, Li L, Yacoub S, Millar JC, et al. Impaired axonal transport contributes to neurodegeneration in a cre-inducible mouse model of myocilin-associated glaucoma. JCI Insight. 2025;10(5). 10.1172/jci.insight.188710 PMC11949003 39836483

[101] Osborne A, Sanderson J, Martin KR. Neuroprotective Effects of Human Mesenchymal Stem Cells and Platelet-Derived Growth Factor on Human Retinal Ganglion Cells. Stem Cells. 2018;36(1):65–78. 29044808 10.1002/stem.2722 PMC5765520

[102] Pham JH, Johnson GA, Rangan RS, Amankwa CE, Acharya S, Stankowska DL. Neuroprotection of rodent and human retinal ganglion cells in vitro/ex vivo by the hybrid small molecule SA-2. Cells. 2022;11(23). 10.3390/cells11233741 PMC9735605 36497005

[103] Harun-Or-Rashid M, Pappenhagen N, Palmer PG, Smith MA, Gevorgyan V, Wilson GN, et al. Structural and Functional Rescue of Chronic Metabolically Stressed Optic Nerves through Respiration. J Neurosci. 2018;38(22):5122–39. 29760184 10.1523/JNEUROSCI.3652-17.2018 PMC5977447

[104] Kim KY, Perkins GA, Shim MS, Bushong E, Alcasid N, Ju S, et al. DRP1 inhibition rescues retinal ganglion cells and their axons by preserving mitochondrial integrity in a mouse model of glaucoma. Cell Death Dis. 2015;6(8):e1839. 26247724 10.1038/cddis.2015.180 PMC4558491

[105] Shim MS, Takihara Y, Kim KY, Iwata T, Yue BY, Inatani M, et al. Mitochondrial pathogenic mechanism and degradation in optineurin E50K mutation-mediated retinal ganglion cell degeneration. Sci Rep. 2016;6:33830. 27654856 10.1038/srep33830 PMC5031982

[106] Harder JM, Guymer C, Wood JPM, Daskalaki E, Chidlow G, Zhang C, et al. Disturbed glucose and pyruvate metabolism in glaucoma with neuroprotection by pyruvate or rapamycin. Proc Natl Acad Sci U S A. 2020;117(52):33619–27. 33318177 10.1073/pnas.2014213117 PMC7776900

[107] Cimaglia G, Tribble JR, Votruba M, Williams PA, Morgan JE. Oral nicotinamide provides robust, dose-dependent structural and metabolic neuroprotection of retinal ganglion cells in experimental glaucoma. Acta Neuropathol Commun. 2024;12(1):137. 39180087 10.1186/s40478-024-01850-8 PMC11342512

[108] Tribble JR, Joe M, Varricchio C, Otmani A, Canovai A, Habchi B, et al. NMNAT2 is a druggable target to drive neuronal NAD production. Nat Commun. 2024;15(1):6256. 39048544 10.1038/s41467-024-50354-5 PMC11269627

[109] Williams PA, Harder JM, Cardozo BH, Foxworth NE, John SWM. Nicotinamide treatment robustly protects from inherited mouse glaucoma. Commun Integr Biol. 2018;11(1):e1356956. 29497468 10.1080/19420889.2017.1356956 PMC5824969

[110] Williams PA, Harder JM, Foxworth NE, Cardozo BH, Cochran KE, John SWM. Nicotinamide and WLD(S) Act Together to Prevent Neurodegeneration in Glaucoma. Front Neurosci. 2017;11:232. 28487632 10.3389/fnins.2017.00232 PMC5403885

[111] Williams PA, Harder JM, John SWM. Glaucoma as a Metabolic Optic Neuropathy: Making the Case for Nicotinamide Treatment in Glaucoma. J Glaucoma. 2017;26(12):1161–8. 28858158 10.1097/IJG.0000000000000767 PMC5854489

[112] De Moraes CG, John SWM, Williams PA, Blumberg DM, Cioffi GA, Liebmann JM. Nicotinamide and Pyruvate for Neuroenhancement in Open-Angle Glaucoma: A Phase 2 Randomized Clinical Trial. JAMA Ophthalmol. 2022;140(1):11–8. 34792559 10.1001/jamaophthalmol.2021.4576 PMC8603231

[113] Cai Q, Jeong YY. Mitophagy in Alzheimer’s disease and other age-related neurodegenerative diseases. Cells. 2020;9(1). 10.3390/cells9010150 PMC7017092 31936292

[114] Kim SH, Munemasa Y, Kwong JM, Ahn JH, Mareninov S, Gordon LK, et al. Activation of autophagy in retinal ganglion cells. J Neurosci Res. 2008;86(13):2943–51. 18521932 10.1002/jnr.21738

[115] Lee SH, Shim KS, Kim CY, Park TK. Characterization of the role of autophagy in retinal ganglion cell survival over time using a rat model of chronic ocular hypertension. Sci Rep. 2021;11(1):5767. 33707562 10.1038/s41598-021-85181-x PMC7952572

[116] Su W, Li Z, Jia Y, Zhuo Y. Rapamycin is neuroprotective in a rat chronic hypertensive glaucoma model. PLoS ONE. 2014;9(6):e99719. 24923557 10.1371/journal.pone.0099719 PMC4055719

[117] Jimenez-Loygorri JI, Benitez-Fernandez R, Viedma-Poyatos A, Zapata-Munoz J, Villarejo-Zori B, Gomez-Sintes R, et al. Mitophagy in the retina: Viewing mitochondrial homeostasis through a new lens. Prog Retin Eye Res. 2023;96:101205. 37454969 10.1016/j.preteyeres.2023.101205

[118] Jimenez-Loygorri JI, Boya P. Aging STINGs: mitophagy at the crossroads of neuroinflammation. Autophagy. 2024;20(7):1684–6. 38411192 10.1080/15548627.2024.2322421 PMC11210893

[119] Lakkaraju A, Boya P, Csete M, Ferrington DA, Hurley JB, Sadun AA, et al. How crosstalk between mitochondria, lysosomes, and other organelles can prevent or promote dry age-related macular degeneration. Exp Eye Res. 2025;251:110219. 39716681 10.1016/j.exer.2024.110219 PMC12097137

[120] Liton PB, Boesze-Battaglia K, Boulton ME, Boya P, Ferguson TA, Ganley IG, et al. Autophagy in the eye: from physiology to pathophysology. Autophagy Rep. 2023;2(1). 10.1080/27694127.2023.2178996 PMC10078619 37034386

[121] Ramirez-Pardo I, Villarejo-Zori B, Jimenez-Loygorri JI, Sierra-Filardi E, Alonso-Gil S, Marino G, et al. Ambra1 haploinsufficiency in CD1 mice results in metabolic alterations and exacerbates age-associated retinal degeneration. Autophagy. 2023;19(3):784–804. 35875981 10.1080/15548627.2022.2103307 PMC9980615

[122] Villarejo-Zori B, Jimenez-Loygorri JI, Zapata-Munoz J, Bell K, Boya P. New insights into the role of autophagy in retinal and eye diseases. Mol Aspects Med. 2021;82:101038. 34620506 10.1016/j.mam.2021.101038

[123] McWilliams TG, Prescott AR, Villarejo-Zori B, Ball G, Boya P, Ganley IG. A comparative map of macroautophagy and mitophagy in the vertebrate eye. Autophagy. 2019;15(7):1296–308. 30786807 10.1080/15548627.2019.1580509 PMC6613837

[124] Gomes C, VanderWall KB, Pan Y, Lu X, Lavekar SS, Huang KC, et al. Astrocytes modulate neurodegenerative phenotypes associated with glaucoma in OPTN(E50K) human stem cell-derived retinal ganglion cells. Stem Cell Rep. 2022;17(7):1636–49. 10.1016/j.stemcr.2022.05.006 PMC9287669 35714595

[125] VanderWall KB, Huang KC, Pan Y, Lavekar SS, Fligor CM, Allsop AR, et al. Retinal Ganglion Cells With a Glaucoma OPTN(E50K) Mutation Exhibit Neurodegenerative Phenotypes when Derived from Three-Dimensional Retinal Organoids. Stem Cell Rep. 2020;15(1):52–66. 10.1016/j.stemcr.2020.05.009 PMC7363877 32531194

[126] Sears NC, Boese EA, Miller MA, Fingert JH. Mendelian genes in primary open angle glaucoma. Exp Eye Res. 2019;186:107702. 31238079 10.1016/j.exer.2019.107702 PMC10207284

[127] Khawaja AP, Cooke Bailey JN, Kang JH, Allingham RR, Hauser MA, Brilliant M, et al. Assessing the Association of Mitochondrial Genetic Variation With Primary Open-Angle Glaucoma Using Gene-Set Analyses. Invest Ophthalmol Vis Sci. 2016;57(11):5046–52. 27661856 10.1167/iovs.16-20017 PMC5040191

[128] Rezaie T, Child A, Hitchings R, Brice G, Miller L, Coca-Prados M, et al. Adult-onset primary open-angle glaucoma caused by mutations in optineurin. Science. 2002;295(5557):1077–9. 11834836 10.1126/science.1066901

[129] Chaphalkar RM, Kodati B, Maddineni P, He S, Brooks CD, Stankowska DL et al. A reduction in mitophagy is associated with glaucomatous neurodegeneration in rodent models of glaucoma. Int J Mol Sci. 2024;25(23). 10.3390/ijms252313040 PMC11642561 39684751

[130] Wei T, Kang Q, Ma B, Gao S, Li X, Liu Y. Activation of autophagy and paraptosis in retinal ganglion cells after retinal ischemia and reperfusion injury in rats. Exp Ther Med. 2015;9(2):476–82. 25574219 10.3892/etm.2014.2084 PMC4280957

[131] Piras A, Gianetto D, Conte D, Bosone A, Vercelli A. Activation of autophagy in a rat model of retinal ischemia following high intraocular pressure. PLoS ONE. 2011;6(7):e22514. 21799881 10.1371/journal.pone.0022514 PMC3142183

[132] Ashrafi G, Schwarz TL. The pathways of mitophagy for quality control and clearance of mitochondria. Cell Death Differ. 2013;20(1):31–42. 22743996 10.1038/cdd.2012.81 PMC3524633

[133] Onishi M, Yamano K, Sato M, Matsuda N, Okamoto K. Molecular mechanisms and physiological functions of mitophagy. EMBO J. 2021;40(3):e104705. 33438778 10.15252/embj.2020104705 PMC7849173

[134] Markaki M, Tsagkari D, Tavernarakis N. Mitophagy mechanisms in neuronal physiology and pathology during ageing. Biophys Rev. 2021;13(6):955–65. 35059020 10.1007/s12551-021-00894-7 PMC8724472

[135] Liu YT, Sliter DA, Shammas MK, Huang X, Wang C, Calvelli H, et al. Mt-Keima detects PINK1-PRKN mitophagy in vivo with greater sensitivity than mito-QC. Autophagy. 2021;17(11):3753–62. 33685343 10.1080/15548627.2021.1896924 PMC8632312

[136] McWilliams TG, Prescott AR, Allen GF, Tamjar J, Munson MJ, Thomson C, et al. mito-QC illuminates mitophagy and mitochondrial architecture in vivo. J Cell Biol. 2016;214(3):333–45. 27458135 10.1083/jcb.201603039 PMC4970326

[137] Hollenbeck PJ. Products of endocytosis and autophagy are retrieved from axons by regulated retrograde organelle transport. J Cell Biol. 1993;121(2):305–15. 7682217 10.1083/jcb.121.2.305 PMC2200099

[138] Hollenbeck PJ, Saxton WM. The axonal transport of mitochondria. J Cell Sci. 2005;118(Pt 23):5411–9. 16306220 10.1242/jcs.02745 PMC1533994

[139] Cheng XT, Zhou B, Lin MY, Cai Q, Sheng ZH. Axonal autophagosomes recruit dynein for retrograde transport through fusion with late endosomes. J Cell Biol. 2015;209(3):377–86. 25940348 10.1083/jcb.201412046 PMC4427784

[140] Miller KE, Sheetz MP. Axonal mitochondrial transport and potential are correlated. J Cell Sci. 2004;117(Pt 13):2791–804. 15150321 10.1242/jcs.01130

[141] Ashrafi G, Schlehe JS, LaVoie MJ, Schwarz TL. Mitophagy of damaged mitochondria occurs locally in distal neuronal axons and requires PINK1 and Parkin. J Cell Biol. 2014;206(5):655–70. 25154397 10.1083/jcb.201401070 PMC4151150

[142] Davis CH, Kim KY, Bushong EA, Mills EA, Boassa D, Shih T, et al. Transcellular degradation of axonal mitochondria. Proc Natl Acad Sci U S A. 2014;111(26):9633–8. 24979790 10.1073/pnas.1404651111 PMC4084443

[143] Harbauer AB, Hees JT, Wanderoy S, Segura I, Gibbs W, Cheng Y, et al. Neuronal mitochondria transport Pink1 mRNA via synaptojanin 2 to support local mitophagy. Neuron. 2022;110(9):1516–e319. 35216662 10.1016/j.neuron.2022.01.035 PMC9081165

[144] Lu B. Neuronal mitophagy: long-distance delivery or eating locally? Curr Biol. 2014;24(20):R1006–8. 25442848 10.1016/j.cub.2014.09.037

[145] Davis CH, Marsh-Armstrong N. Discovery and implications of transcellular mitophagy. Autophagy. 2014;10(12):2383–4. 25484086 10.4161/15548627.2014.981920 PMC4502649

[146] Wang X, Winter D, Ashrafi G, Schlehe J, Wong YL, Selkoe D, et al. PINK1 and Parkin target Miro for phosphorylation and degradation to arrest mitochondrial motility. Cell. 2011;147(4):893–906. 22078885 10.1016/j.cell.2011.10.018 PMC3261796

[147] Syc-Mazurek SB, Libby RT. Axon injury signaling and compartmentalized injury response in glaucoma. Prog Retin Eye Res. 2019;73:100769. 31301400 10.1016/j.preteyeres.2019.07.002 PMC6898776

[148] Kim M, Sandford E, Gatica D, Qiu Y, Liu X, Zheng Y, et al. Mutation in ATG5 reduces autophagy and leads to ataxia with developmental delay. Elife. 2016;5. 10.7554/eLife.12245 PMC4786408 26812546

[149] Waetzig R, Matthes M, Leister J, Penkivech G, Heise T, Corbacioglu S, et al. Comparing mTOR inhibitor Rapamycin with Torin-2 within the RIST molecular-targeted regimen in neuroblastoma cells. Int J Med Sci. 2021;18(1):137–49. 33390782 10.7150/ijms.48393 PMC7738968

[150] Vershinina YS, Krasnov GS, Garbuz DG, Shaposhnikov MV, Fedorova MS, Pudova EA, et al. Transcriptomic analysis of the effect of torin-2 on the central nervous system of drosophila melanogaster. Int J Mol Sci. 2023;24(10). 10.3390/ijms24109095 PMC10219255 37240439

[151] Kasetti RB, Maddineni P, Millar JC, Clark AF, Zode GS. Increased synthesis and deposition of extracellular matrix proteins leads to endoplasmic reticulum stress in the trabecular meshwork. Sci Rep. 2017;7(1):14951. 29097767 10.1038/s41598-017-14938-0 PMC5668243

[152] Kasetti RB, Maddineni P, Patel PD, Searby C, Sheffield VC, Zode GS. Transforming growth factor beta2 (TGFbeta2) signaling plays a key role in glucocorticoid-induced ocular hypertension. J Biol Chem. 2018;293(25):9854–68. 29743238 10.1074/jbc.RA118.002540 PMC6016452

[153] Kasetti RB, Patel PD, Maddineni P, Patil S, Kiehlbauch C, Millar JC, et al. ATF4 leads to glaucoma by promoting protein synthesis and ER client protein load. Nat Commun. 2020;11(1):5594. 33154371 10.1038/s41467-020-19352-1 PMC7644693

[154] Kasetti RB, Patel PD, Maddineni P, Zode GS. Ex-vivo cultured human corneoscleral segment model to study the effects of glaucoma factors on trabecular meshwork. PLoS ONE. 2020;15(6):e0232111. 32579557 10.1371/journal.pone.0232111 PMC7314024

[155] Maddineni P, Kasetti RB, Zode GS. Methods for Analyzing Endoplasmic Reticulum Stress in the Trabecular Meshwork of Glaucoma Models. Methods Mol Biol. 2018;1695:121–34. 29190024 10.1007/978-1-4939-7407-8_12

