# Supplementary material for: Pharmacological restoration of impaired autophagy in retinal ganglion cells prevents abnormal mitochondrial accumulation and glaucomatous neurodegeneration
Source: Mol Neurodegener. 2026 May 16;21:30. doi: 10.1186/s13024-026-00950-4 (PMC13242679; doi:10.1186/s13024-026-00950-4)
Supplement: Supplementary file 1 — Supplementary Material 1 [file 13024_2026_950_MOESM1_ESM.docx]

**Supplementary Figures and Figure Legends:**

**
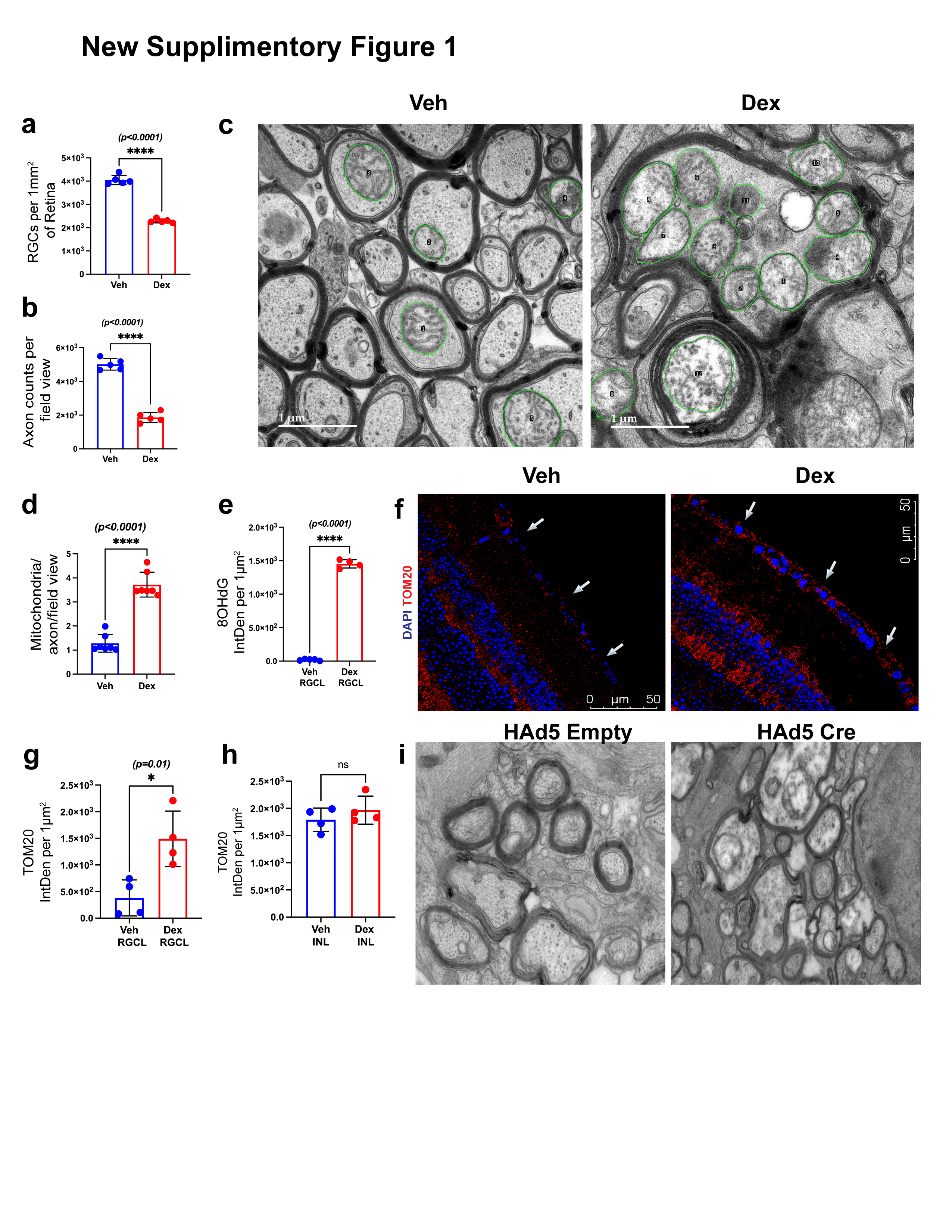
**

**Supplementary Figure 1: OHT leads to accumulation of dysfunctional mitochondria.**

**a & b)** Mice injected with Dex for 10 weeks show a significant reduction in the number of RBPMS-positive RGCs and healthy axons compared with Veh-injected mice, respectively (n = 5 per group). **c**) Representative TEM images showing mitochondria within ON axons from 10-week Veh- and Dex-injected eyes. Mitochondria (green circles) were traced using ImageJ for quantitative analysis of mitochondrial number, area, and circumference. **d**) Quantification of the total number of mitochondria per axon from 10-week Veh- and Dex-injected eyes (n = 7). **e**) Quantitative analysis of 8-OHdG staining. **f -h**) TOM20 expression and its quantitative analysis in the RGCL and INL of Dex-induced OHT eyes compared with Veh-injected eyes, respectively (n = 4). **i)** Representative TEM images of ON cross sections from *Tg.Cre-MYOC^Y437H^* mice injected with HAd5-Cre or HAd5-empty vectors (n = 4 per group).


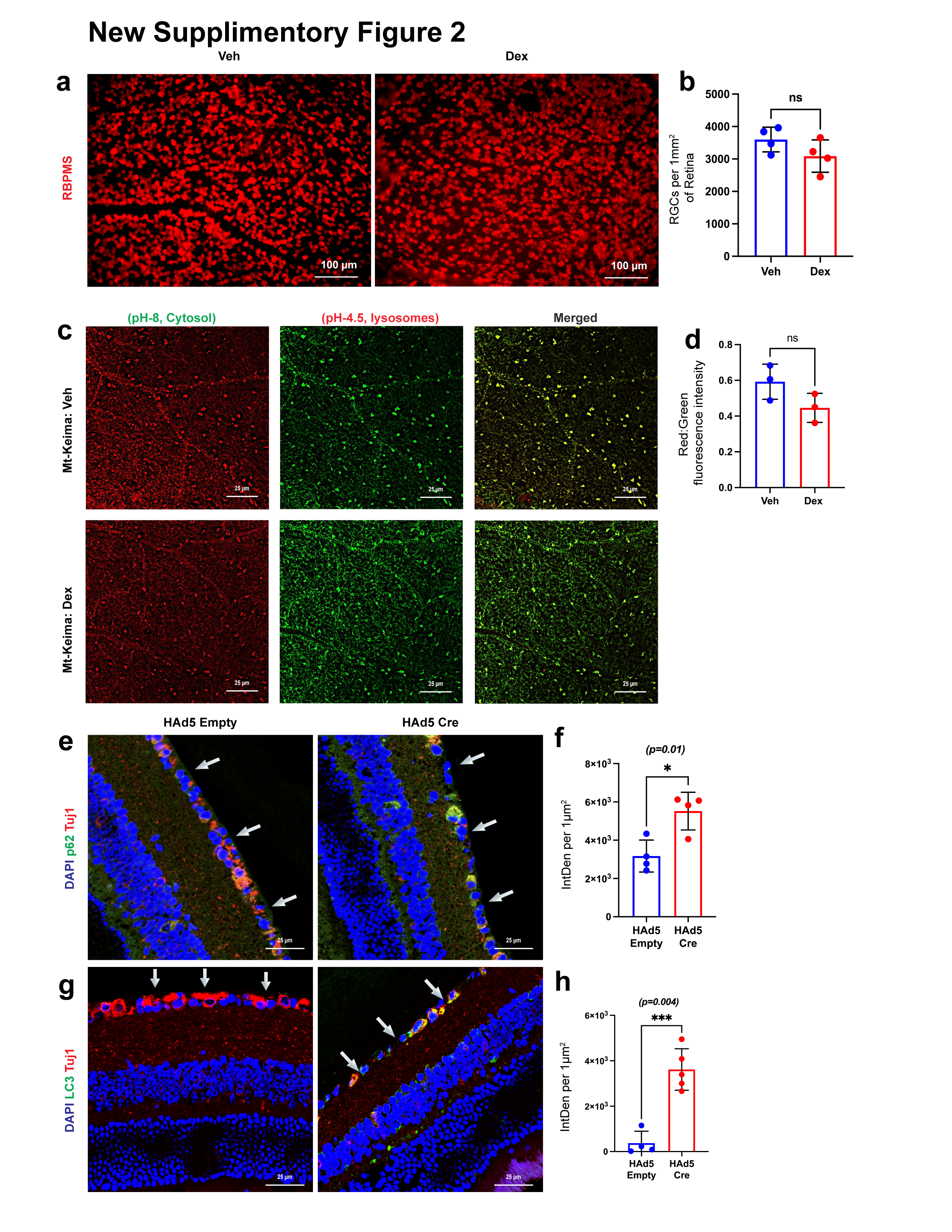


**Supplementary Figure 2: OHT leads to impaired autophagy. a**) Representative whole-mount retinal images stained with RBPMS and **b**) the corresponding dot plots showed no significant RGC loss between the 5-week Veh- and Dex-injected mice (n = 4 in each group). **c & d**) Mitophagy flux was assessed in flat-mounted retinas from Mt-Keima reporter mice injected with Veh or Dex for 5 weeks. A non-significant reduction in mitophagy flux was observed in Dex-injected OHT eyes compared to contralateral Veh-injected eyes (n = 3 in each group). Immunostaining and quantitative analyses showed increased expression of **e & f)** p62 and **g & h**) LC3B in the RGCL of HAd5-Cre-injected *Tg.Cre-MYOC^Y437H^* mice compared to HAd5-empty-injected *Tg.Cre-MYOC^Y437H^* littermates (n = 4-5 in each group; arrows indicate RGCL).

**
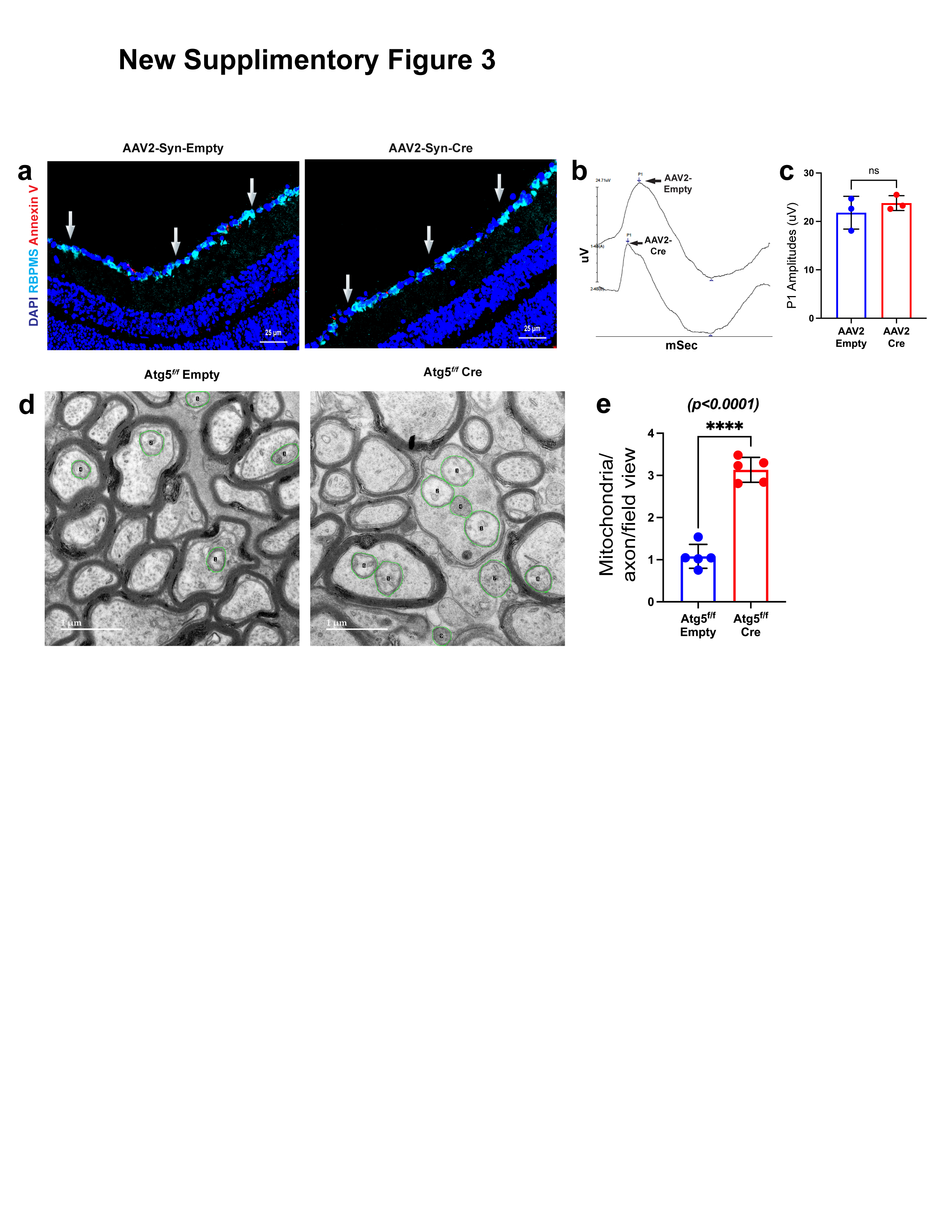
**

**Supplementary Figure 3: Impaired autophagy leads to damaged mitochondrial accumulation.** C57BL/6J wild-type mice were intravitreally injected with AAV2-Syn-empty in one eye and AAV2-Syn-Cre in the contralateral eye. Representative **a**) retinal cross-sections immunostained with Annexin V and **b & c**) PERG amplitudes showed no significant difference between the eyes. **d**) TEM images showing mitochondria within optic nerve (ON) axons from *Atg5^flox/flox^* mice injected with AAV2-empty or AAV2-Syn-Cre. Mitochondria (green circles) were traced using ImageJ software for quantitative analysis of their number, area, and circumference. e) Quantification of the total number of mitochondria per axon from *Atg5^flox/flox^* mice injected with AAV2-empty or AAV2-Syn-Cre (n = 5).

**
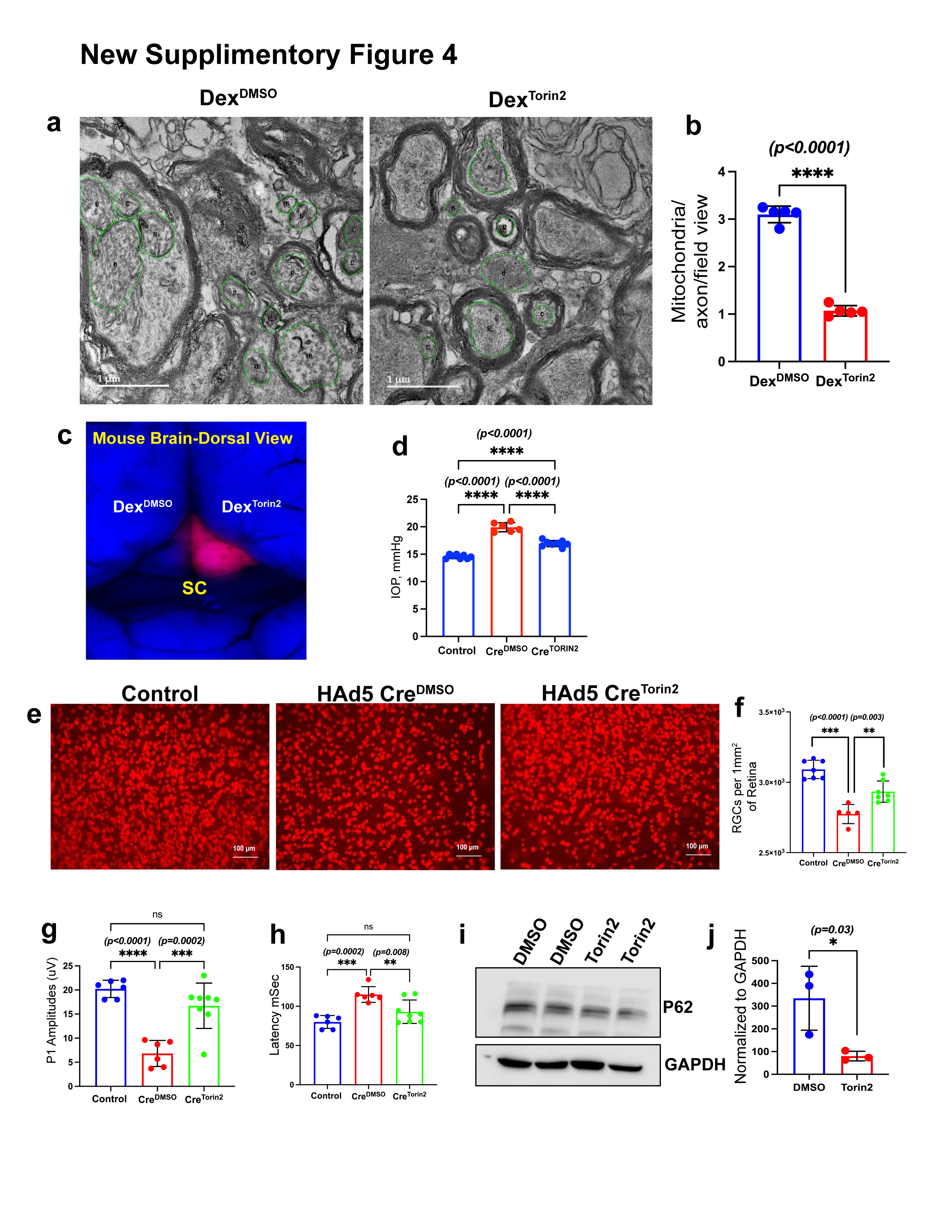
**

**Supplementary Figure 4: Torin 2 treatment prevents glaucomatous neurodegeneration in mouse models of glaucoma. a**) Representative TEM images showing mitochondria within ON axons from 10-week Dex-injected OHT eyes treated with DMSO or Torin 2. Mitochondria (green circles) were traced using ImageJ software for quantitative analysis of their number, area, and circumference. **b**) Quantification of the total number of mitochondria per axon in 10-week Dex-injected OHT eyes treated with DMSO or Torin 2 (n = 5). **c**) Anterograde transport of CTB to the superior colliculus in the mouse brain in 10-week Dex-injected OHT eyes treated with DMSO or Torin 2 (n = 3). d-h) In the *Tg.Cre-MYOC^Y437H^* glaucoma model, Torin 2 treatment (**d**) reduced IOP, (**e & f**) prevented RGC loss, and (**g & h**) improved pERG amplitudes (n = 7-8 per group). **I & j**) Ex vivo retinal explants treated with Torin 2 for 12 hrs showed reduced p62 expression compared with controls (n = 3).
