# Supplementary figures and images for: Pharmacological restoration of impaired autophagy in retinal ganglion cells prevents abnormal mitochondrial accumulation and glaucomatous neurodegeneration
Source: Mol Neurodegener. 2026 May 16;21:30. doi: 10.1186/s13024-026-00950-4 (PMC13242679; doi:10.1186/s13024-026-00950-4)

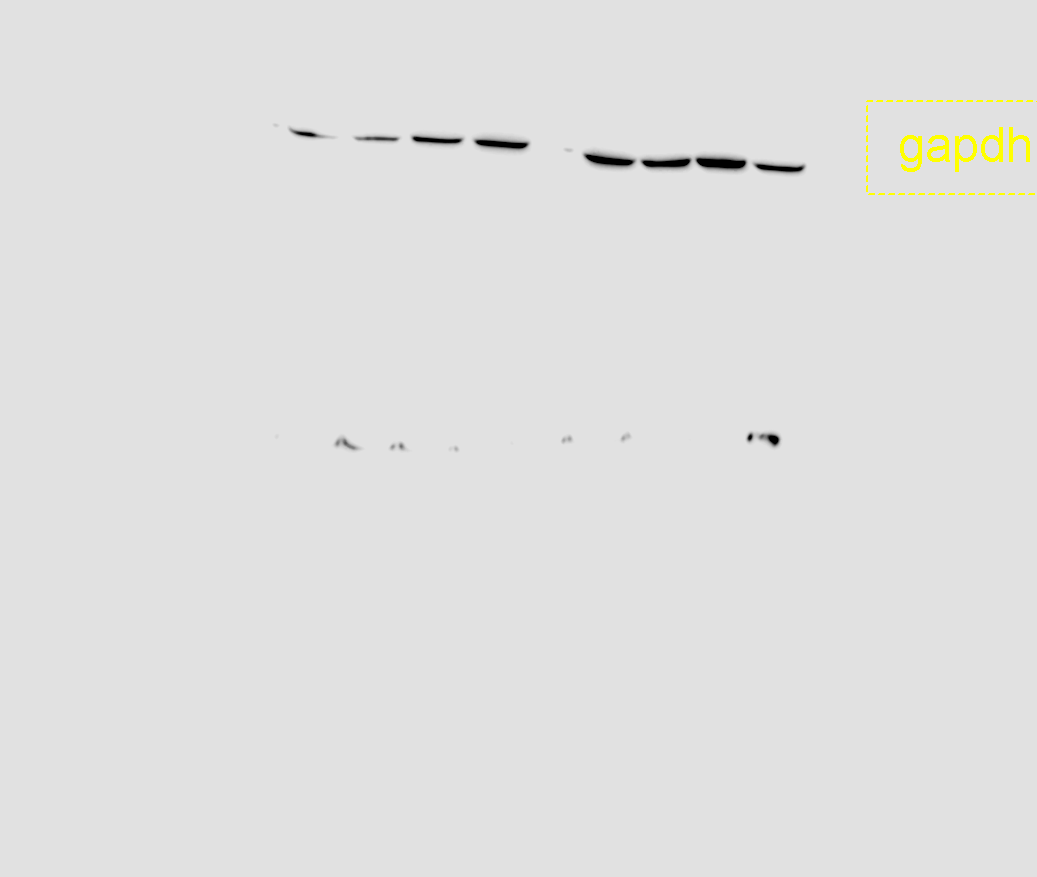

Supplement: Supplementary file 2 — Supplementary Material 2 [file 13024_2026_950_MOESM2_ESM.tif]

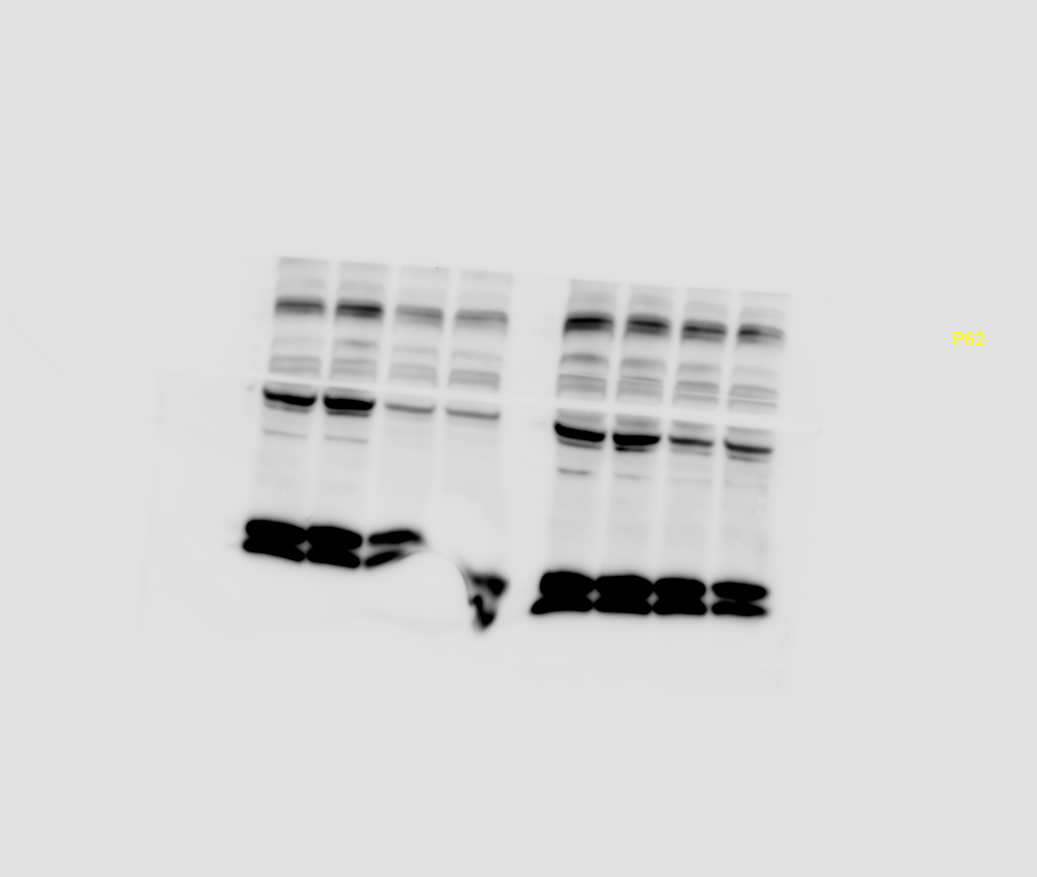

Supplement: Supplementary file 3 — Supplementary Material 3 [file 13024_2026_950_MOESM3_ESM.tif]
